# Visible-light-driven reactions for the synthesis of sulfur dioxide-inserted compounds: generation of S–F, S–O, and S–N bonds

**DOI:** 10.1039/d3ra02067c

**Published:** 2023-05-11

**Authors:** Truong Giang Luu, Hee-Kwon Kim

**Affiliations:** a Department of Nuclear Medicine, Jeonbuk National University Medical School and Hospital Jeonju 54907 Republic of Korea hkkim717@jbnu.ac.kr; b Research Institute of Clinical Medicine of Jeonbuk, National University-Biomedical Research, Institute of Jeonbuk National University Hospital Jeonju 54907 Republic of Korea

## Abstract

Sulfur dioxide-containing compounds such as sulfonyl fluorides, sulfonyl esters, and sulfonyl amides are important structural frameworks in many natural products, pharmaceuticals, and organic compounds. Thus, synthesis of these molecules is a very valuable research topic in organic chemistry. Various synthetic methods to introduce SO_2_ groups into the structure of organic compounds have been developed for the synthesis of biologically and pharmaceutically useful compounds. Recently, visible-light-driven reactions were carried out to create SO_2_-X (X = F, O, N) bonds, and their effective synthetic approaches were demonstrated. In this review, we summarized recent advances in visible-light-mediated synthetic strategies for generation of SO_2_-X (X = F, O, N) bonds for various synthetic applications along with proposed reaction mechanisms.

## Abstract 2

A review of recent advances in visible-light-mediated synthetic strategies for generation of SO_2_-X (X = F, O, N) bonds to give sulfonyl compounds.

## Graphical abstract

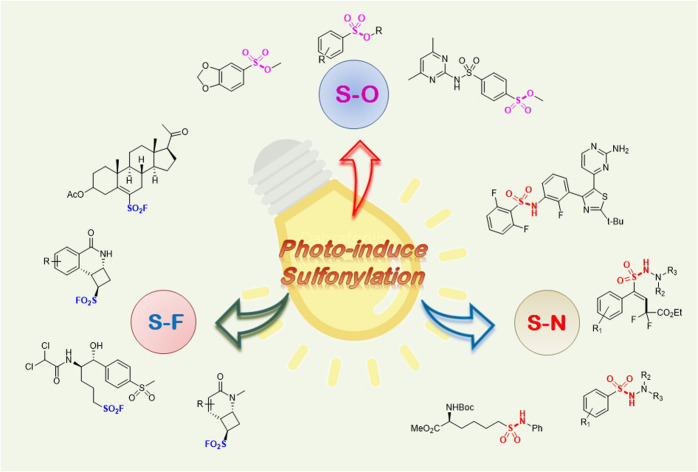

## 1. Introduction

**Fig. 1:**
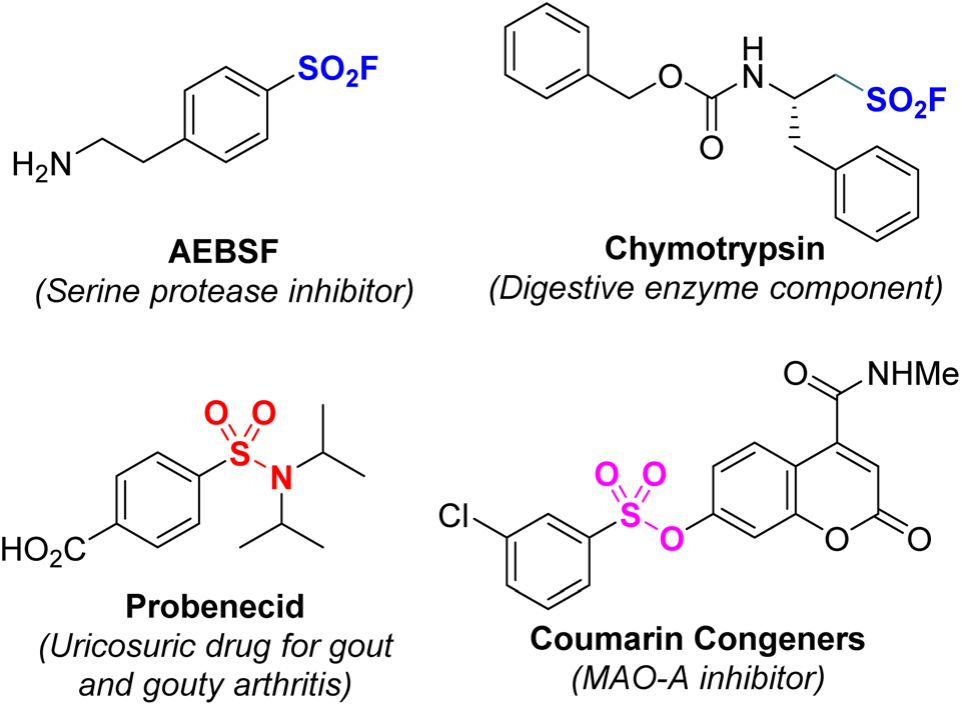
Bioactive compounds containing sulfonyl groups.

Sulfur is a common non-metallic element in the natural environment and living organisms. In particular, sulfur-containing organic compounds often play an important role in biological processes.^1–3^ For example, sulfonyl fluoride has been shown to be a useful compound with wide applications in medical sciences, proteomics, and materials science.^4–9^ Sulfonamides have many applications such as antihypertensive drugs; nonsteroidal anti-inflammatory drugs; and as diuretics, anticancer and antibacterial agents, and anticonvulsants.^10–15^ Sulfone esters are widely employed as anticancer agents, antimicrotubule agents, MAO-A inhibitors, and phosphor-STAT3 inhibitors.^16–20^ In addition, they are also considered high-value building blocks used in organic synthesis and pharmaceutical chemistry (Fig. 1).^21,22^

The good biological activity of the sulfone molecules has attracted scientific attention. In particular, compounds bearing SO_2_-X (X = F, O, N) bonds have attracted much attention.^23,24^ Traditionally used methods for synthesis of sulfone derivatives have generally focused on inserting sulfur-containing groups such as thiols and disulfide into organic molecules, followed by sulfide oxidation.^25–27^ However, these processes required the use of strong oxidizing agents as well as harsh reaction conditions that are incompatible with sensitive functional groups. This has limited the scope of the substrates as well as their practical applications, including that in pharmaceutical synthesis.^28,29^ Recently, several studies have analyzed direct introduction of SO_2_ into organic molecules.

The SO_2_ function group is a very effective moiety for trapping carbon radicals, and the unsaturated bonds are attractive targets for sulfonyl radicals. Therefore, generation of new sulfonyl radicals is an interesting approach to build various SO_2_.^30–40^ Diverse sources of sulfonyl groups have been found, and SO_2_ can be obtained from various sources such as sulfonic acid, sulfonate inorganic salts, sulfonyl halides, sulfonyl hydrazide, and DABSO.^41–46^

Since visible-light-mediated chemical reactions were first discovered, photochemistry has proven a useful strategy in several fields including chemical synthesis and pharmaceutical chemistry.^47–53^ Photochemical reactions have several advantages over conventional synthesis reactions including low cost, use of renewable energy, and non-hazardous redox reagents.^54–58^ Photoreactions can also be conducted under mild conditions, which help increase their safety and promote applications.

With these advantages, photochemical reactions are a suitable method for various processes. These approaches have also provided novel and creative synthetic studies and can be applied to wide chemical reactions of compounds bearing sensitive functional groups.^59–61^ Recently, photochemical reactions have been used for generation of sulfonic fluorides, sulfonic esters, and sulfonamides.

Herein, we summarize the developments of visible light-driven reactions for synthesis of compounds with SO_2_-X (X = F, O, N) bonds using several strategies.

## 2. S–X bond formation reaction

### 2.1. Visible light induced synthesis of sulfonyl fluorides (S–F bond formation)

In recent years, sulfonyl fluorides have received much attention because of their unique properties and wide utilization. In addition to applications in pharmaceutical chemistry^62–64^ and materials,^65,66^ they are also considered as potential candidates to replace sulfonyl chloride in organic synthesis processes.^67–69^ Sharpless and co-workers first reported sulfur(vi) fluoride exchange (SuFEx), and sulfonyl fluorides were employed for novel generation of “click chemistry”.^70–72^ Therefore, a series of photochemical reactions to produce sulfonyl fluorides have been reported.

**Scheme 1:**
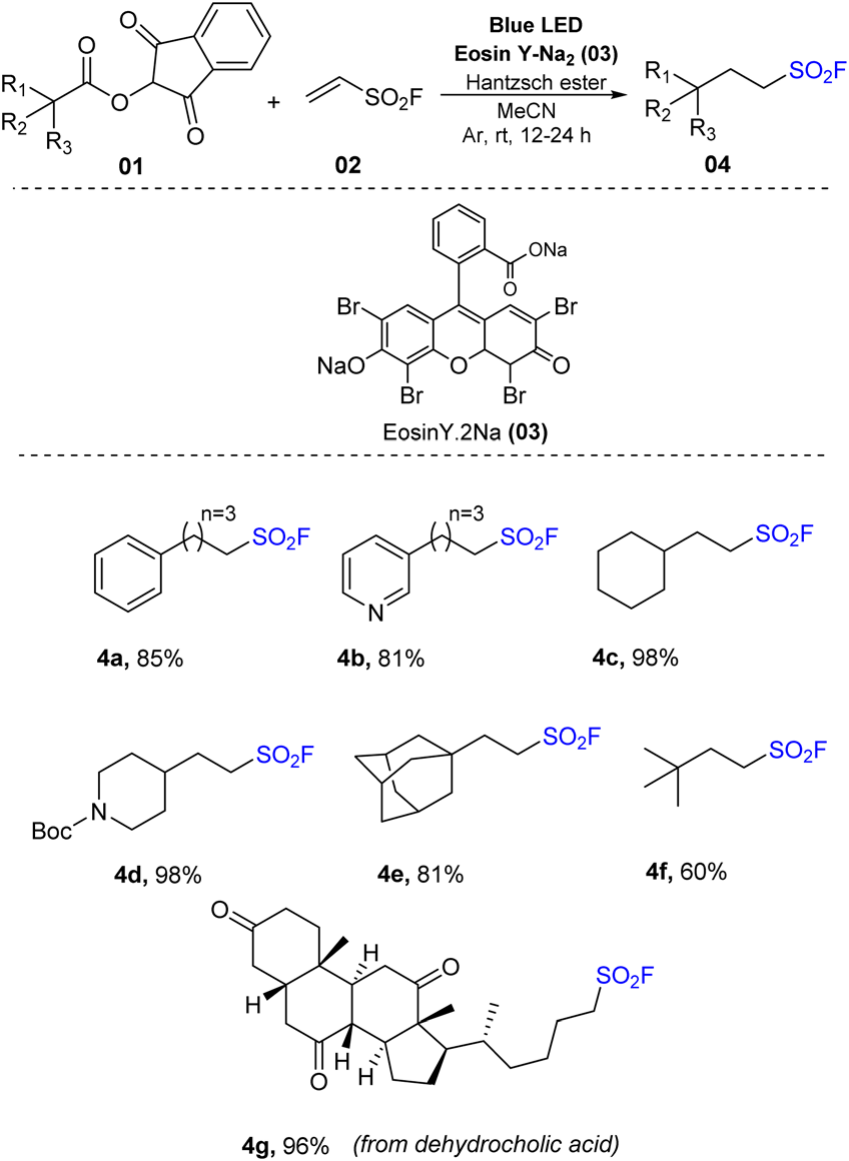
Synthesis of aliphatic sulfonyl fluorides *via* decarboxylation.

In 2019, Liao and co-workers reported a facile method to produce aliphatic sulfonyl fluoride structures through decarboxylation (Scheme 1).^73^ The synthesis of sulfonyl fluorides was achieved *via* reaction between *N*-hydroxyphthalimide (NHPI) esters, which could provide alkyl radicals, and vinyl sulfonyl fluoride (VSF). The reactions were conducted in the presence of eosin Y as a photocatalyst and Hantzsch ester (HE) in MeCN under irradiation of blue LED. This method was designed to use abundant sources of carboxylic acids to produce sulfonyl fluoride products. This method tolerated numerous carboxylic acids including primary, secondary, and tertiary acids with different functional groups to produce the corresponding products in high yields. Using the process, secondary acids such as cyclohexane carboxylic acid were converted to the desired products (4c) with outstanding yields (98%). Similarly, pyridine-4-carboxylic acid with a Boc protecting group was used to give sulfonyl fluoride (4d) under photo-reaction conditions. Tertiary acids including pivalic acid and adamantane acid reacted well and were transformed to the corresponding products (4e and 4f) in good yields (60–81%). Several drugs with acid functional groups have also been tested to evaluate the applicability of the method to synthesis of pharmaceuticals, and reaction of dehydrocholic acids with vinyl sulfonyl fluoride could produce the target product (4g) with high yield (96%).

**Scheme 2:**
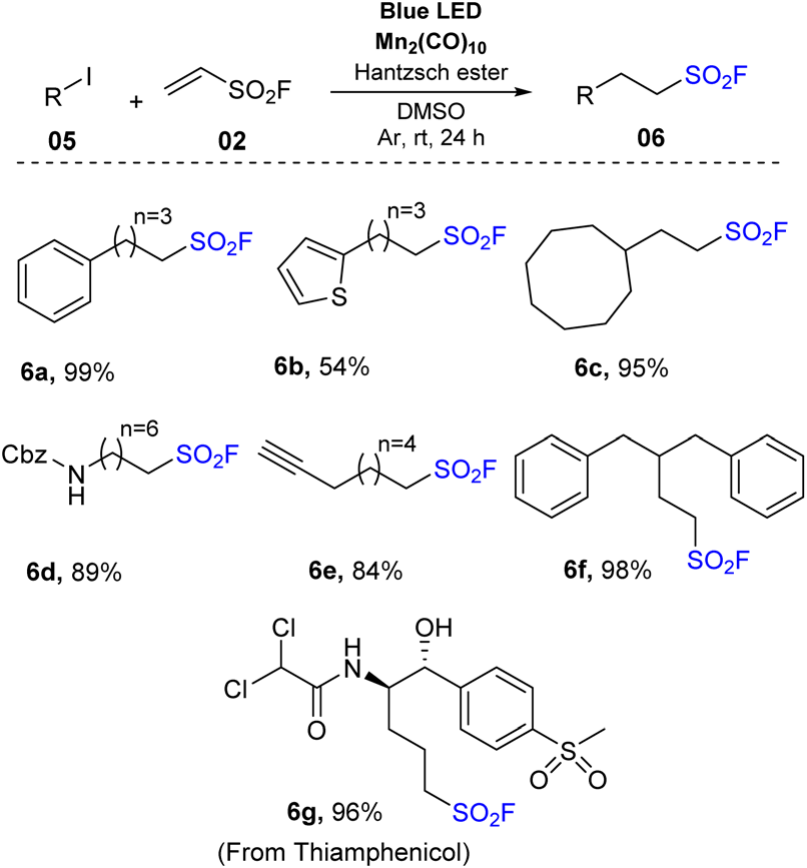
Synthesis of sulfonyl fluorides *via* reductive addition of alkyl iodides to ethenesulfonyl fluoride.

Another synthesis method of aliphatic sulfonyl fluoride derivatives using source SO_2_F (generated directly from vinyl sulfonyl fluoride) was reported by Qin and co-workers in 2021 (Scheme 2).^74^ In their study, alkyl radicals were obtained directly from alkyl halides instead of having pre-activation of carboxylic acids as NHPI esters. To prepare sulfonyl fluorides, alkyl halides were reacted with vinyl sulfonyl fluoride in the presence of Mn_2_(CO)_10_ as a photocatalyst and Hantzsch ester as a reductant in DMSO under irradiation of blue LED at room temperature. Using this method, primary alkyl iodides bearing functional groups such as aryl(hetero) groups, acyclic long chains, and benzyl carbamate moiety were smoothly converted to the corresponding products (6a–6f) with moderate to good yields. Interestingly, favorable transformation of benzyl iodide to the target product (6a) was achieved with a 99% yield. Secondary alkyl iodides including various alicyclic rings and (hetero)aryl groups were tolerated in the reaction to provide sulfonyl fluoride compounds (6b, 6c) in 54–98% yields. For tertiary alkyl iodides, products were prepared with significantly lower yields than primary and secondary alkyl iodines under the same reaction conditions. Several other alkyl halides such as alkyl chlorides and alkyl bromides have also been employed using this developed method, but no products were formed. Additionally, many derivatives of natural products and drug molecules were tested. Target sulfonyl fluoride derivative (6g) was successfully prepared with a high yield, providing the wide applicability of this method.

**Scheme 3:**
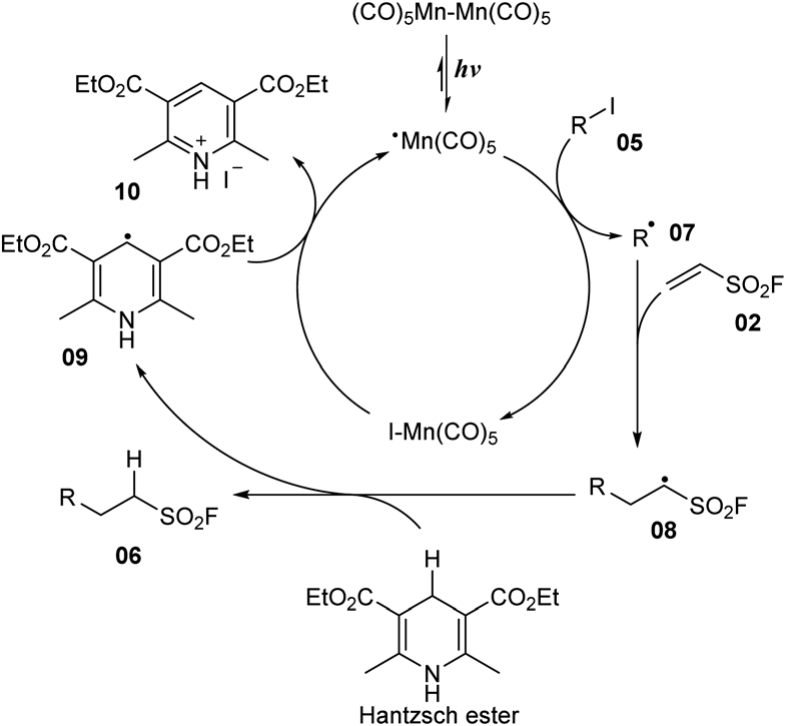
A plausible mechanism of radical mediated alkyl sulfonylation.

A mechanism was proposed as shown in Scheme 3. Photocatalyst Mn_2_(CO)_10_ participated in Mn–Mn bond homolytic cleavage to generate [˙Mn(CO)_5_] radical under irradiation of visible-light. Then, [˙Mn(CO)_5_] radical reacted with iodine substrate 05 to provide alkyl radical 07 and I–Mn(CO)_5_ compound. Alkyl radical 07 further attacked the double bond of vinyl sulfonyl fluoride 02 to afford new alkyl sulfonyl fluoride radical 08, which captured one proton from the Hantzsch ester to form desired product 06 and radical 09. Finally, the I–Mn(CO)_5_ compound interacted with radical 09 to return [˙Mn(CO)_5_] radical and salt iodine 10.

**Scheme 4:**
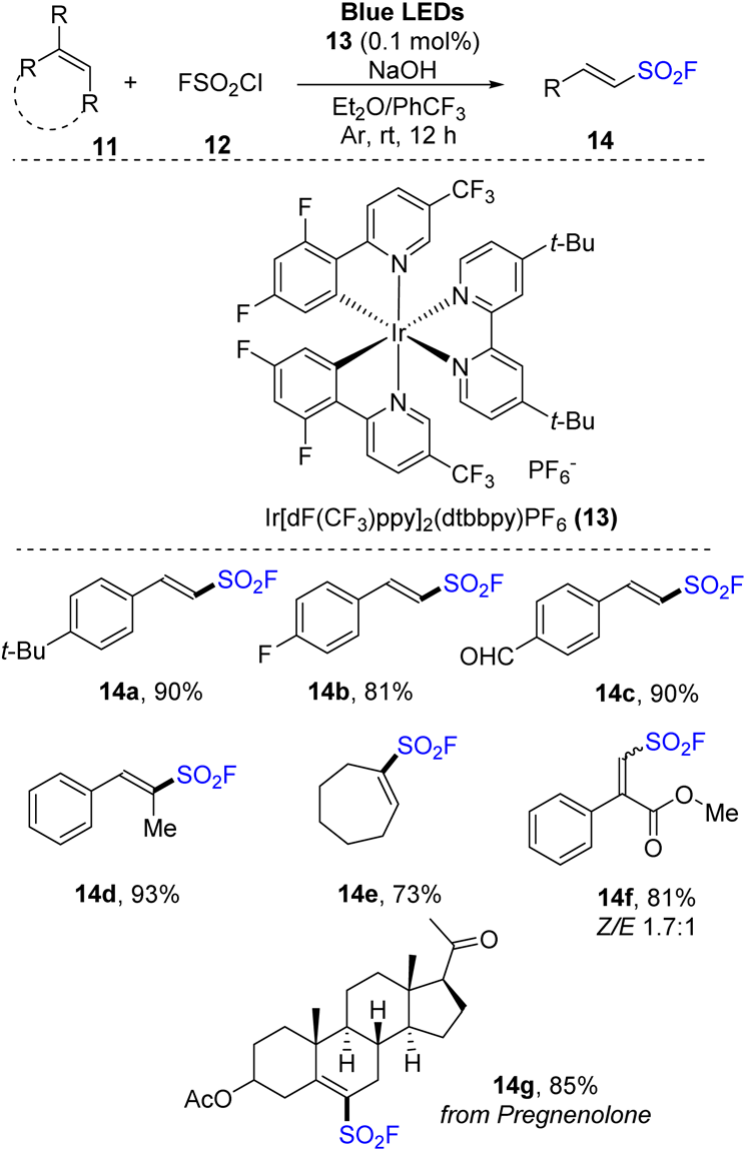
Radical fluorosulfonylation of olefins.

Most syntheses of sulfonyl fluorides use SO_2_F^+^ cation as a fluoride reagent. However, fluorosulfonyl radical (FSO_2_˙) reagents were also used. In 2021, Liao and co-workers developed a method for synthesis of alkenyl sulfonyl fluorides using FSO_2_ precursors that effectively released FSO_2_ radicals.^75^ Various alkenes were reacted with sulfuryl chlorofluoride (SO_2_F) as a radical source in the presence of Ir[dF(CF_3_)ppy]_2_(dtbbpy)PF_6_ as a photocatalyst and with NaOH in a mixture of diethyl ether and PhCF_3_ under irradiation of blue LED (Scheme 4). The reactions tolerated a wide range of phenyl alkene derivatives with both electron-donating and electron-withdrawing groups. Reactions of substrates with various functional groups such as aldehyde, ketone, ester, and nitro groups smoothly produced the corresponding sulfonyl fluoride derivatives (14a–14c). Using the process, substrates with many structures such as heterocycles, naphthalene, and amines were also converted into the desired products with high yields. One benefit of this approach is to achieve the direct synthesis of special structures of sulfonyl fluorides (14d–14f) from fluorosulfonylate cyclic, di-, and tri-substituted olefins, which are difficult to synthesize using SO_2_F^+^ cations. A natural product (14g) also was successfully converted to its corresponding product by this process.

**Scheme 5:**
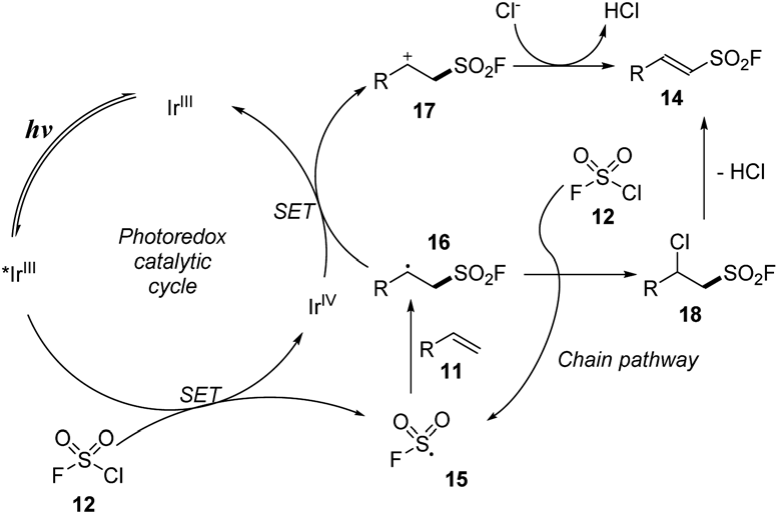
A possible reaction pathway for radical fluorosulfonylation of olefins.

The proposed mechanism for the method is shown in Scheme 5. Under irradiation of visible light, the photo-process of Ir^III^ catalyst provided an excited *Ir^III^, which participated in the single-electron transfer process with chlorosulfonyl fluoride 12 to form FSO_2_ radical 15 and Ir^IV^. FSO_2_ radical 15 reacted with alkene 11 to make a new radical at C-center 16. The C-center radical 16 underwent a single electron transfer (SET) process with Ir^IV^ to return Ir^III^, generating the C-center cation 17, which lost a proton to the Cl^−^ anion to form the product 14. In another pathway, the C-center radical 16 reacted directly with chlorosulfonyl fluoride 12 to form chloride intermediate 18, which then released an HCl molecule to form the target product 14.

**Scheme 6:**
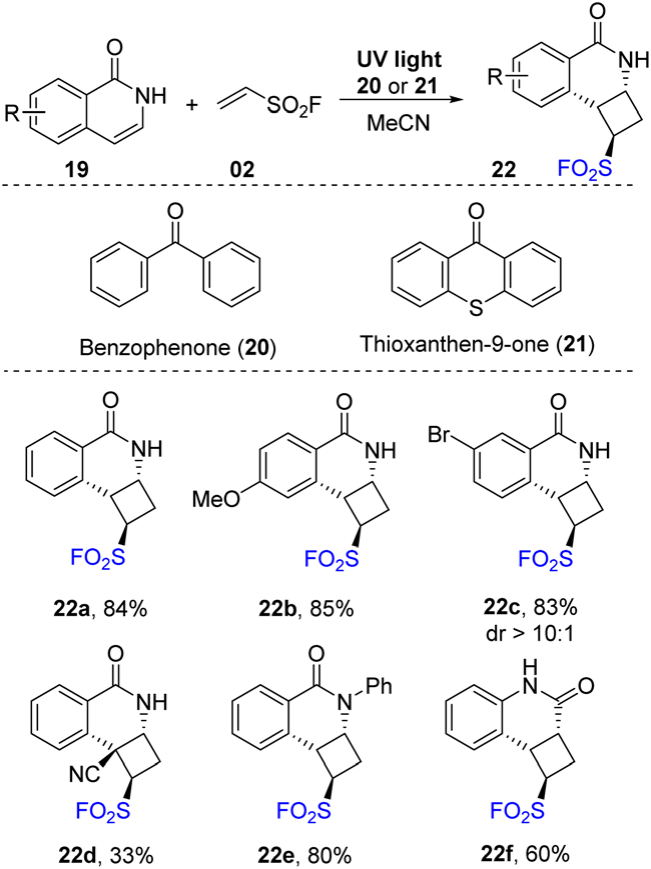
[2 + 2] Photocycloaddition reaction of isoquinolones for assembly of cyclobutanes bearing both pyridinyl and sulfonyl fluoride functionalities.

In 2020, Qin and co-workers described the synthesis of cyclobutanes containing pyridinyl and sulfonyl fluoride groups.^76^ In their study, [2 + 2] photocycloaddition reactions were conducted in the presence of isoquinolones or pyridones as pyridinyl substrates and ethenesulfonyl fluoride as a SO_2_F source in MeCN under irradiation of UV light. In specific cases, benzophenone or thioxanthen-9-one was used as a photocatalyst (Scheme 6). For derivatives of isoquinolones, both naked and substituent carrier substrates were successfully employed to give the corresponding products with good yields. In the reactions, the reaction efficiencies were not significantly affected by substituents including electron-withdrawing and electron-donating groups in different positions (22a–22c) on the aromatic ring. A substrate with a 4-cyano functional group was transformed to the desired sulfonyl fluoride 22d with 33% yield. Additionally, *N*-substituted isoquinolone and quinolone substrates were tolerated with this method to afford the products (22e, 22f) in high yields (60–80%). Notably, while almost products were provided as single isomers, several products were formed with diastereomeric ratio from 20 : 1 to 10 : 1.

**Scheme 7:**
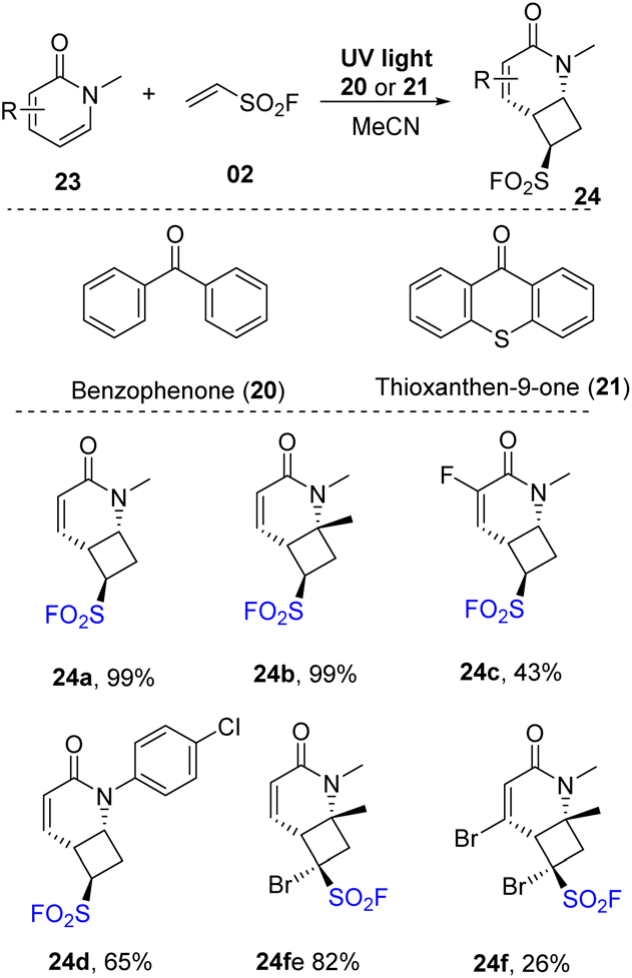
[2 + 2] Photocycloaddition reaction of pyridones for assembly of cyclobutanes bearing both pyridinyl and sulfonyl fluoride functionalities.

Pyridones, which have skeletons similar to isoquinolones, were investigated for this protocol (Scheme 7). Most pyridone derivatives with electron-donating groups underwent this process to form products (24a, 24b) with outstanding yields (up to 99%). Reaction with substrates bearing electron-withdrawing groups provided the corresponding products (24c) with lower yields. Several *N*-substituents were tolerated in the reaction to generate the desired sulfonyl fluoride 24d. 1-Bromoethene-1-sulfonyl fluoride (instead of vinyl sulfonyl fluoride) was used in this [2 + 2] cycloaddition reaction, and most of the reactions were smoothly carried out to provide the corresponding cycloaddition products (24e, 24f).

**Scheme 8:**
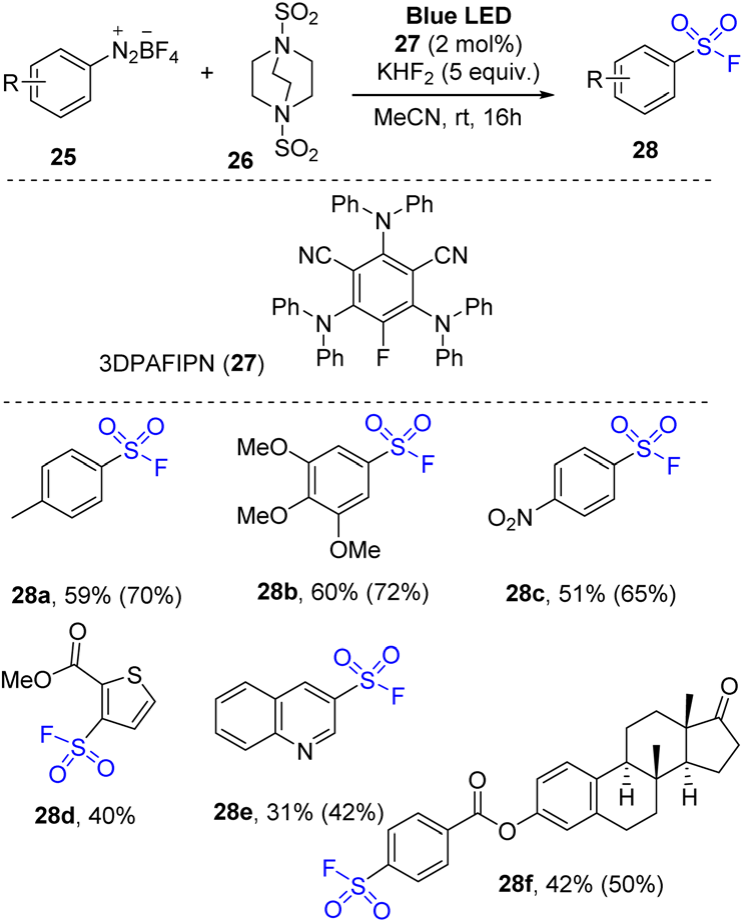
Synthesis to arylsulfonyl fluorides using aryldiazonium salts.

Aryl diazonium salts were employed for organo-photoredox reaction to synthesize arylsulfonyl fluorides by Tlili and co-workers in 2021.^77^ The reactions were conducted through a one-pot multicomponent reaction using arylazo tetrafluoroborate salts as a starting material, DABSO as a sulfonyl substrate, KHF_2_ as a fluoride source, and 3DPAFIPN as a photocatalyst in MeCN under irradiation of a blue LED at room temperature (Scheme 8). Various arylazo derivatives were successfully employed for the reaction to provide the products. Photoreactions of arene substrate with electron-donating groups were performed, and the desired arylsulfonyl fluorides (*e.g.*, methyl-substituted arylsulfonyl fluorides (28a)) were obtained with moderate yield. The trimethoxyphenyl substitute product (28b) was obtained with 60% yield, suggesting that steric hindrance did not significantly affect the reaction outcome. Additionally, the electron-poor substitute substrates (*e.g.*, nitro derivatives) were converted to the corresponding products (28c). Heterocyclic substrates were tolerated in the reaction to afford the desired sulfonyl fluorides (28d and 28e). To evaluate potential application of the reaction, a complex molecular architecture (compound 28f) was tested and successfully yielded the target products in a moderate yield.

**Scheme 9:**
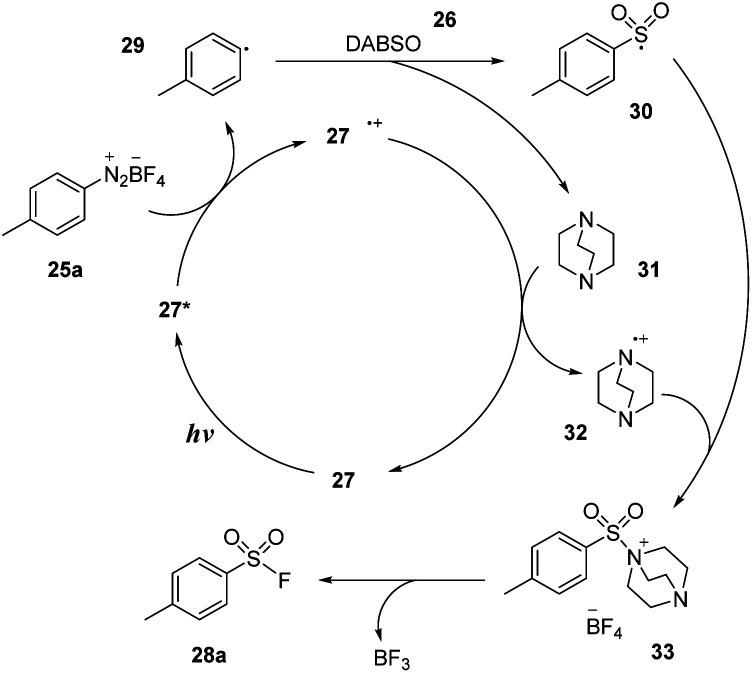
Proposed mechanism for synthesis of arylsulfonyl fluorides.

A plausible mechanism was proposed based on EPR spectroscopy as well as DFT calculations as shown in Scheme 9. Photocatalyst 27 was excited and transformed to 27*, which reduced diazonium salt 25a through the SET process to generate aryl radical 29 and (27˙^+^) cation radical. Aryl radical 29 reacted with DABSO 26 to form aryl sulfonyl radical 30 and DABCO 31. Then, DABCO 31 interacted with (27˙^+^) cation radical to return the ground state photocatalyst 27 and (DABCO˙^−^) anion radical 32. Anion radical 32 combined with sulfonyl radical 30 to provide intermediate salt 33. Finally, a nucleophilic fluorine attack generated the desired product 28a and reformed DABCO 31 and BF_3_.

**Scheme 10:**
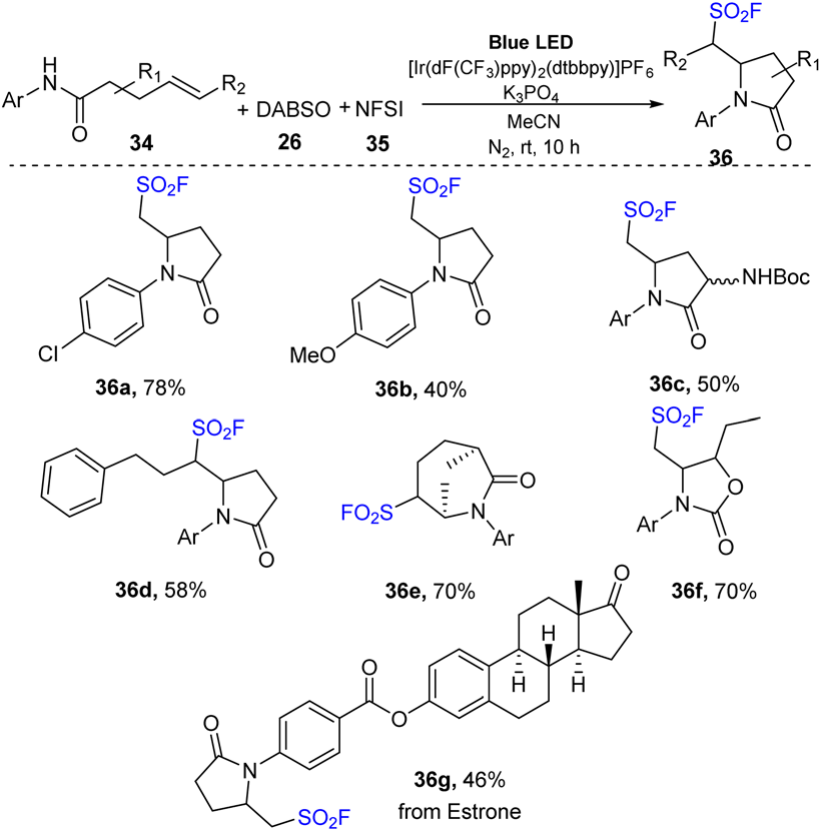
Aminofluorosulfonylation of unactivated alkenes by merging photocatalytic PCET activation.

Direct attack on double bonding of alkenes is a useful and effective strategy for affording complex and diverse products. In 2021, Weng and co-workers reported a multi-component reaction to yield an amino-fluoro sulfonyl structure by combining a proton-coupled electron transfer (PCET) process and a radical relay process (Scheme 10).^78^ In this method, *N*-aryl pent-4-enamides were reacted with DABSO and NSFI in the presence of [Ir(dF(CF_3_)ppy)_2_(dtbbpy)]PF_6_ as a photocatalyst and K_3_PO_4_ as a base in MeCN under irradiation of a blue LED at room temperature. Reaction of substrates with electron-withdrawing groups at the *p*-position of the *N*-aryl groups smoothly provided the corresponding product 36a, and substrates with electron-donating groups were transformed to sulfonyl fluoride 36b with lower yield. Products bearing an *N*-heteroaryl amide moiety were prepared with high yields. Reactions of substrates with various substituents at the double bond of olefins were also investigated. Terminal olefin substrates with different substituents such as benzyl or amino groups with protecting groups as well as nonterminal olefin substrates were smoothly converted to the corresponding sulfonyl fluoride products (36c, 36d) in good yields. This reaction of compounds containing an endocyclic double bond proceeded smoothly to afford complexes with fused polycyclic structures (36e). In addition, substrates from drugs or natural compounds were well transformed to target sulfonyl fluoride products including sulfamethazine, an antibacterial agent, and estrone derivative (36f, 36g).

**Scheme 11:**
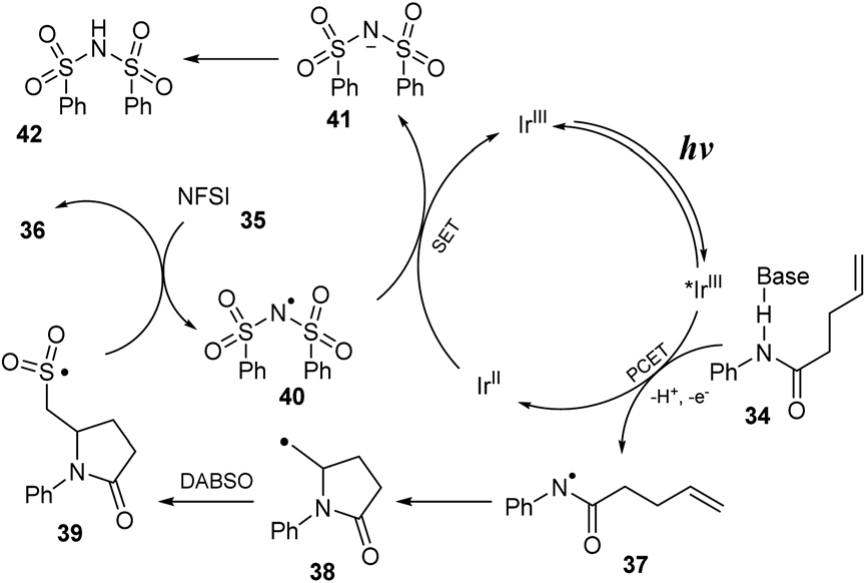
Proposed mechanism of the three-component aminofluorosulfonylation reaction.

A proposed mechanism of this reaction is shown in Scheme 11. The excited *Ir^III^ was formed from ground state Ir^III^ under visible light irradiation. Then, it underwent a proton-coupled electron transfer (PCET) process with substrate 34 to provide *N*-central radical 37 and Ir^II^. Next, radical 37 participated in closing the 5-sided ring to generate C-central radical 38, which further reacted with DABSO to provide sulfonyl radical 39. In the final step, sulfonyl radical 39 captured F^−^ anion from NSFI to afford the product 36 and (PhSO_2_)_2_N radical 40 that interacted with Ir^II^ to return Ir^III^ and (PhSO_2_)_2_N anion 41. After that, (PhSO_2_)_2_N anion 41 received a proton to produce (PhSO_2_)_2_NH as a byproduct.

**Scheme 12:**
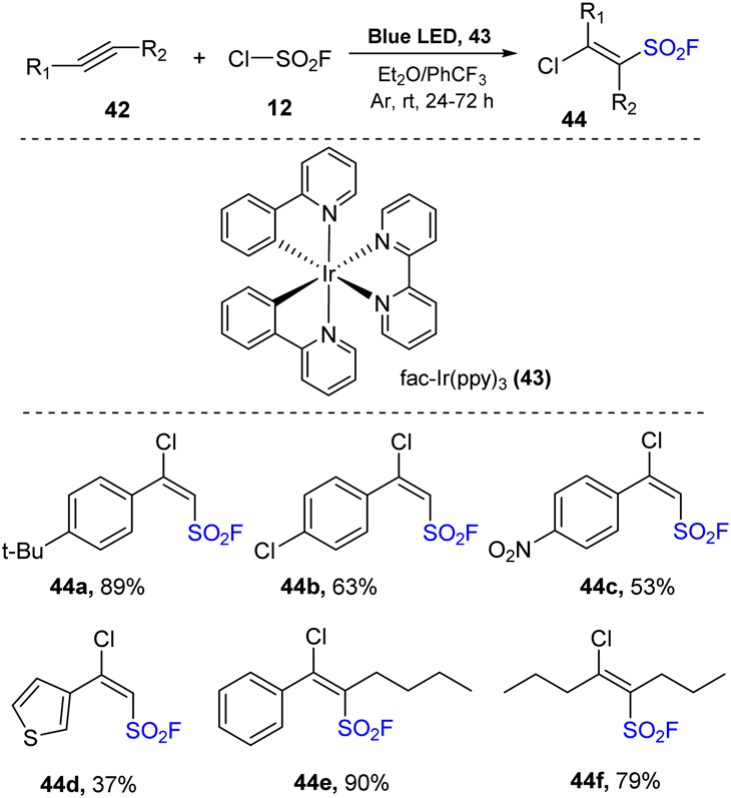
Chloro-fluorosulfonylation of alkynes.

On previous study, FSO_2_Cl has been shown to be an efficient FCO_2_ radical source for organic synthesis.^75^ With same strategy, Liao and co-workers developed of the synthesis of β-chloro alkenyl sulfonyl fluorides (BCASF) as novel sulfonyl fluoride hubs by using FSO_2_Cl in 2021.^79^ Diverse BCASFs were prepared *via* treatment of alkynes with FSO_2_Cl in the presence of *fac*-Ir(ppy)_3_ as a photocatalyst in a mixture of Et_2_O and PhCF_3_ under irradiation of blue LEDs at room temperature for 24 to 72 hours (Scheme 12). Alkenyl sulfonyl fluoride products containing various functional groups including electron-donating 44a and halide 44b groups on benzene rings were generated in moderate to good yields using the method. Substrates bearing electron-withdrawing groups were smoothly transformed into the products 44c because of the strong electrophilicity of FSO_2_ radicals. When a heterocyclic substrate was employed, target sulfonyl fluoride 44d was prepared in 37% yield. Additionally, internal alkynes easily underwent this process to form the desired alkenyl sulfonyl fluorides 44e in 90% yield. This protocol was successfully applied to reactions of aliphatic alkynes to generate the corresponding products 44f (79%).

**Scheme 13:**
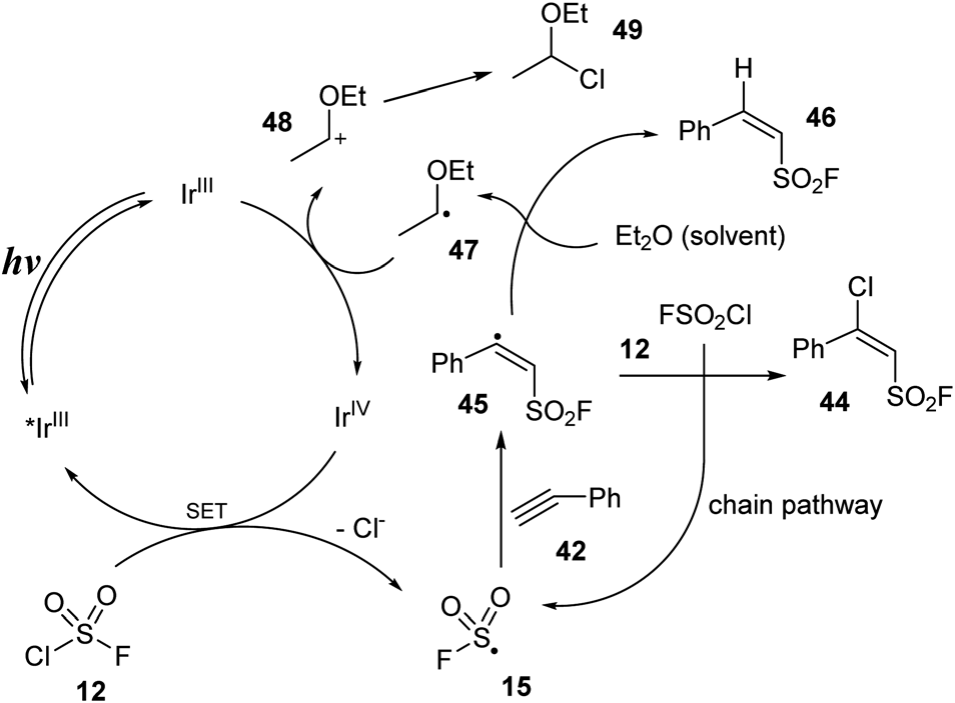
A possible reaction pathway for chloro-fluorosulfonyl difunctionalization of alkynes.

A proposed mechanism for this method is illustrated in Scheme 13. Under irradiation of blue LEDs, iridium catalyst Ir^III^ was excited to the *Ir^III^ state. A single-electron transfer (SET) process between the excited iridium *Ir^III^ and FSO_2_Cl 12 was conducted to provide FSO_2_ radical 15, chloride anion Cl^−^, and Ir^IV^. FSO_2_ radical 15 attacked the triple bond of alkyne 42 to generate radical 45. This radical received one chloride atom from FSO_2_Cl 12 to form desired product 44 and recovered FSO_2_ radical 15. Radical 45 was also involved in the hydrogen-atom transfer (HAT) process with Et_2_O to give byproduct 46 and intermediate 47, which further transferred one electron to iridium catalyst Ir^IV^ to give the iridium catalyst at ground-state Ir^III^ and cation 48. 48 reacted with chloride anion Cl^−^ to form byproduct 49.

**Scheme 14:**
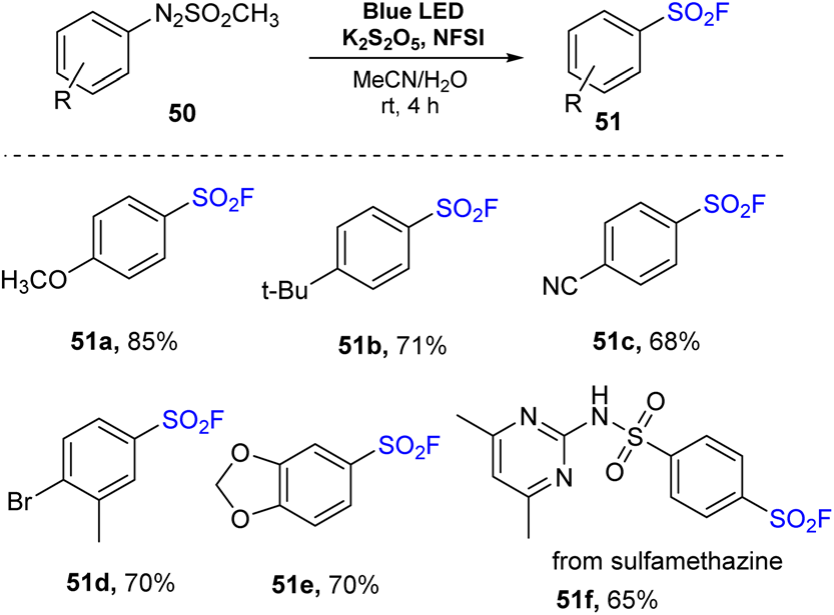
Visible-light-mediated sulfonylfluorination of arylazo sulfones.

In 2022, Kim and co-workers performed a novel sulfonylfluorination from arylazo sulfones.^80^ In this method, arylazo sulfones were reacted with K_2_S_2_O_5_ and NSFI in a mixture of MeCN and H_2_O under irradiation of visible light at room temperature without any photocatalyst to prepare sulfonyl fluorides (Scheme 14). Reactions of naked arylazo sulfones as well as substrates bearing electron-donating groups such as methoxy and *tert*-butyl groups were readily carried out to give the corresponding products (51a and 51b) with high yields. Substrates bearing electron-withdrawing groups at the *p*-position were also utilized to give the desired sulfonyl fluoride 51c. Using this process, substrates containing various functional groups such as di-substitutes and heterocycles were smoothly converted to the target products (51d and 51e) in good yields. When the arylazo sulfone created from the antibacterial medication sulfamethazine was employed in the reaction, the desired sulfonyl fluoride 51f was obtained with 65% yield.

**Scheme 15:**
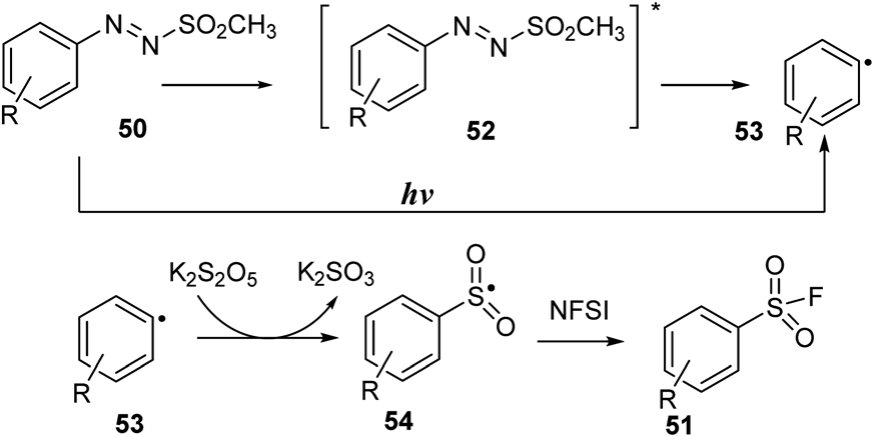
Proposed mechanism for synthesis of aryl sulfonyl fluorides.

A possible reaction mechanism is shown in Scheme 15. Homolytic cleavage of the N–S bond of arylazo sulfone 50 by irradiation of visible light gave aryl radical 53. Then, aryl radical 53 was reacted with K_2_S_2_O_5_, an SO_2_ source agent, to create arylsulfonyl radical 54. A fluorine atom transfer from NFSI to aryl sulfonyl radical 54 afforded aryl sulfonyl fluoride 51.

**Scheme 16:**
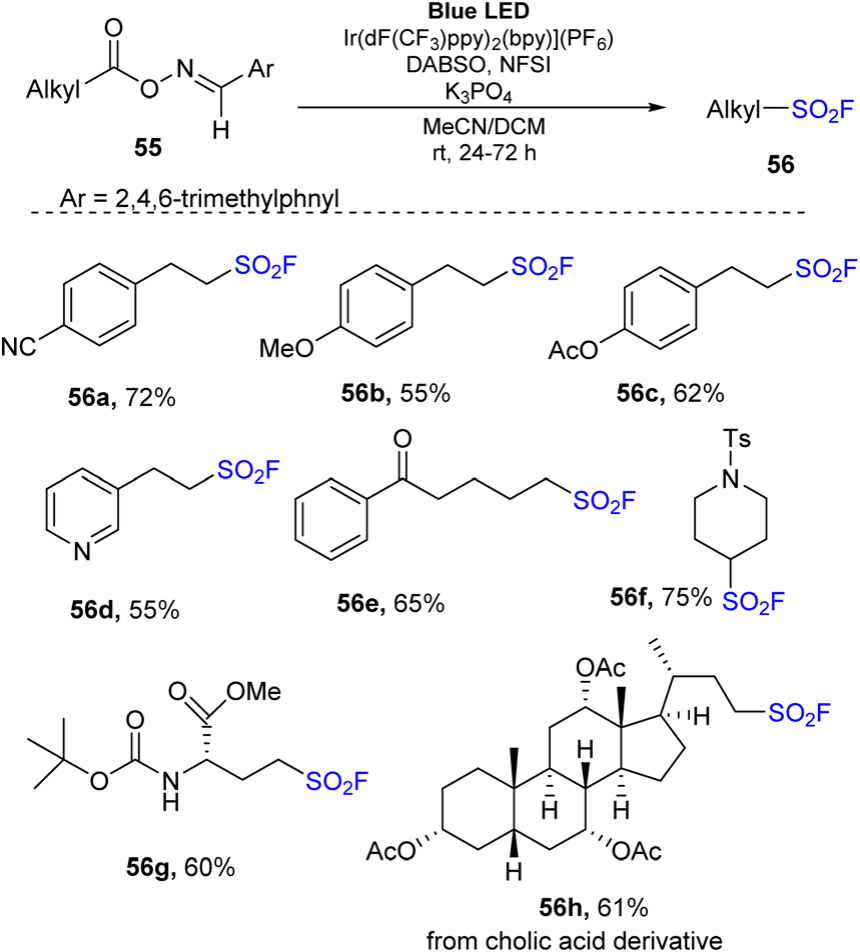
Decarboxylative fluorosulfonylation with oxime esters of carboxylic acids.

In 2022, Weng and co-workers carried out decarboxylative fluorosulfonylation of aldoxime esters of aliphatic carboxylic acid.^81^ Aldoxime esters were reacted with DABSO and NSFI in the presence of Ir(dF(CF_3_)ppy)_2_(bpy)](PF_6_) as a photocatalyst and K_3_PO_4_ as a base in a mixture of MeCN and CH_2_Cl_2_ under irradiation of a blue LED at room temperature for 24 h (Scheme 16). Oxime esters generated from 3-aryl propionic acids bearing electron-donating groups or electron-withdrawing groups were tolerated in the reaction to generate the corresponding compounds (56a and 56b) in good yields. Sensitive substituents such as esters were tested in the reaction, and they were unaffected by the process, generating the desired product 56c. Using the reaction, oxime esters of heteroaryl, benzoyl, and secondary carboxylic acids were smoothly transformed to the corresponding sulfonyl fluorides (56d, 56e, and 56f) in 55–75% yields. Reaction using oxime ester generated for amino acid 56g readily yielded the target product in 60% yield. Oxime esters of cholic acid delivered the corresponding sulfonyl fluoride 56h with 66% yield.

**Scheme 17:**
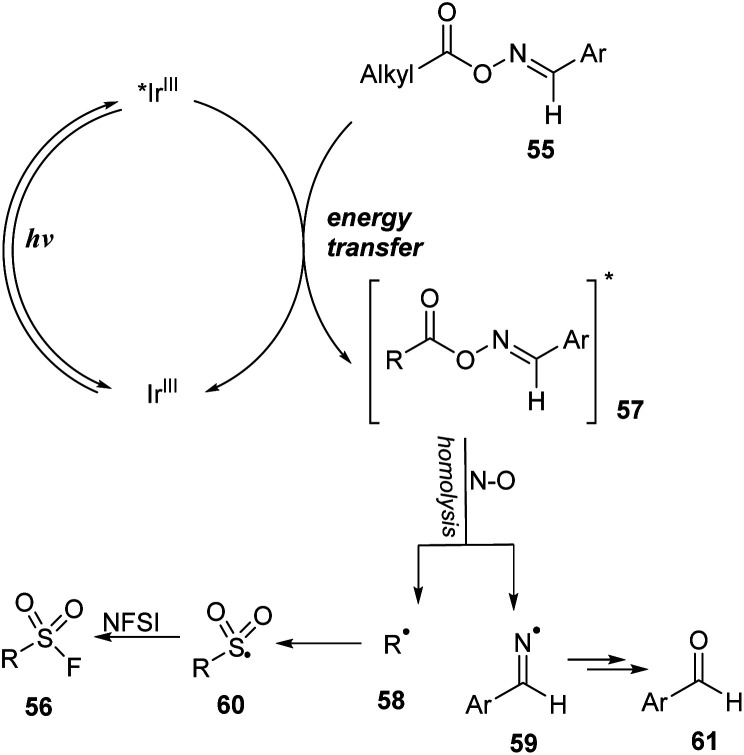
Plausible mechanism for decarboxylative fluorosulfonylation with oxime esters of carboxylic acids.

A mechanism for this method was proposed as shown in Scheme 17. Photocatalyst Ir^III^ was excited to *Ir^III^ under irradiation of a blue LED. Then, it underwent the energy transfer process with oxime ester substrate 55 to form the excited oxime ester 57 and the ground state Ir^III^. In the excited state, the oxime ester 57 was rapidly decomposed by homo-cleavage of an N–O bond to generate alkyl radical 58 and iminyl radical 59. Alkyl radical 58 further received a SO_2_ molecule and F atom, respectively, from DABSO and NSFI to form the target product 56.

**Scheme 18:**
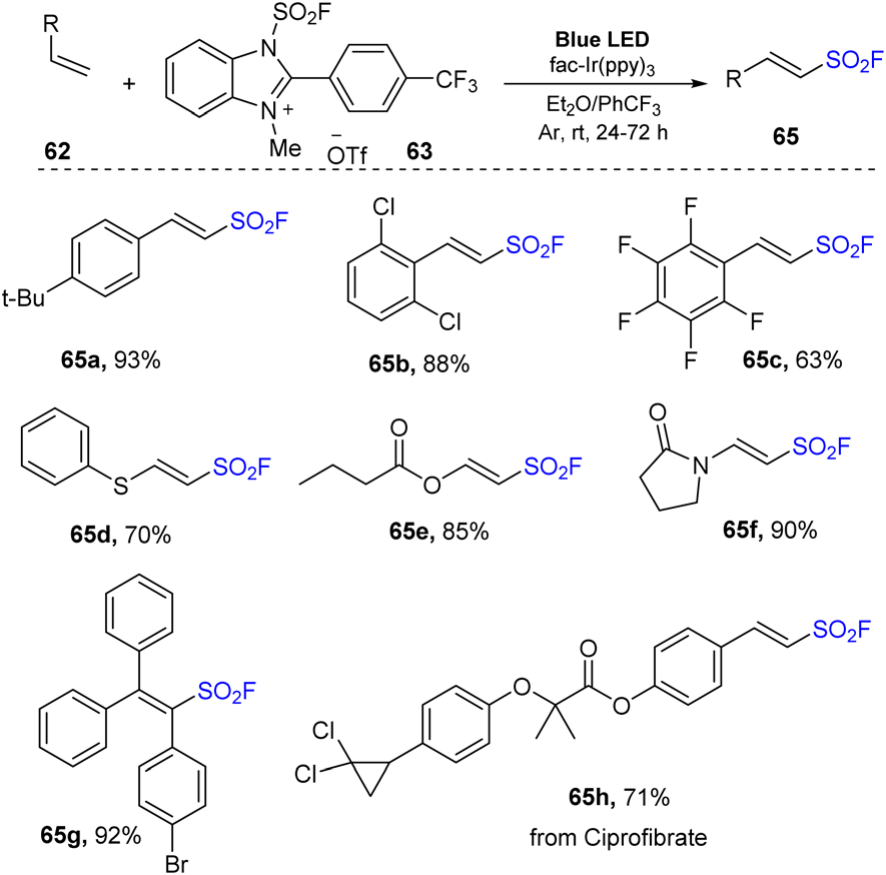
Radical fluorosulfonylation of multi-substituted olefins and natural products.

In 2022, Liao and co-workers reported fluorosulfonylation of alkenes using 1-fluorosulfonyl 2-aryl benzoimidazolium triflate (FABI) salts that gave a SO_2_F group as a radical (Scheme 18).^82^ Reactions between olefins and FABI salts were conducted in the presence of *fac*-Ir(ppy)_3_ as a photocatalyst in 1,4-dioxane under irradiation of blue LED at room temperature for 12 hours. Regardless of the amount, type, and position of the substituents attached to the benzene ring, alkene substrates were readily converted to the corresponding products (65a–65c) with good to excellent yields. However, because of their lower oxidation potential, aliphatic alkene reactions provided the desired products with less efficiency than that of styrene derivatives. This method worked well with electron-rich olefins to produce vinyl sulfonyl fluorides that were *O*- (66e) or *N*-substituted (66f). Using the reaction, multi-substituted products including cyclic, di- and tri-substituted vinyl sulfonyl fluorides (66g) were smoothly obtained in good yields.

**Scheme 19:**
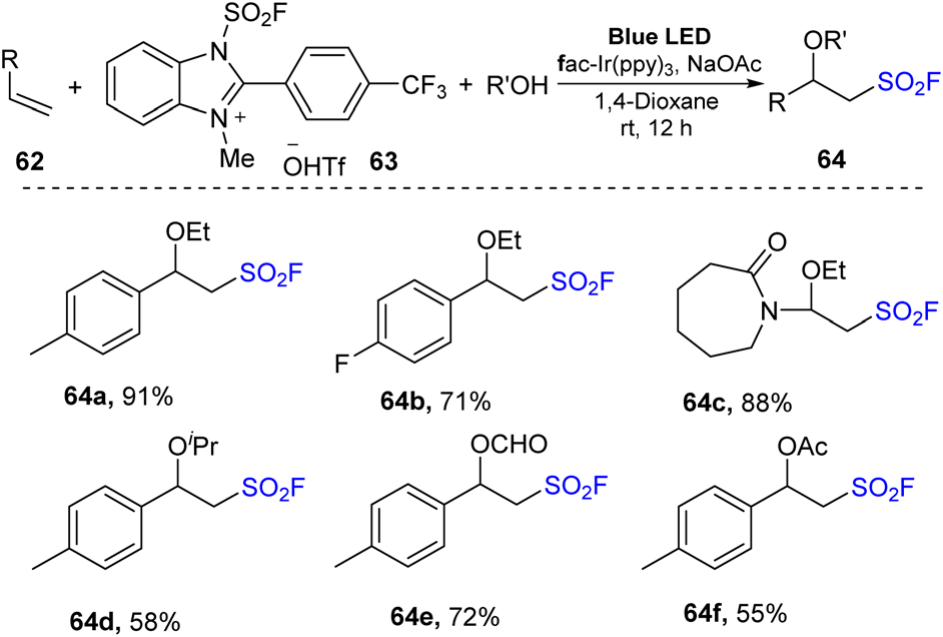
Radical fluorosulfonylation of terminal olefins and electron-rich alkenes.

In an extensive study, alkoxy-fluorosulfonyl difunctionalization reactions of olefins were conducted in the presence of alcohols as nucleophiles (Scheme 19). Most styrene derivatives and electron-rich olefins were reacted with alcohols such as methanol and isopropanol to deliver the desired products (64a–64f) in moderate to good yields.

**Scheme 20:**
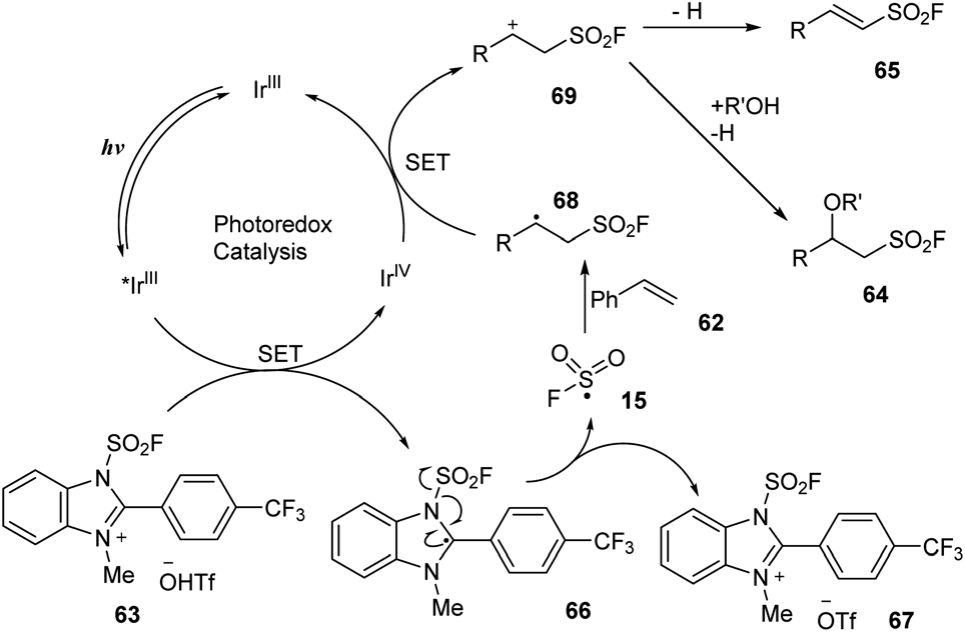
Possible reaction mechanism for radical fluorosulfonylation using FABI.

A proposed mechanism of this reaction is shown in Scheme 20. Photocatalyst Ir^III^ was excited under irradiation of visible light and underwent a SET process with FABI salt 63 to create Ir^IV^ and compound 66. After receiving one electron, 66 participated in homo-cleavage of the N–S bond to form FSO_2_ radical 15, which attacked olefin 62 to form intermediate radical 68. Oxidation of 68 by Ir^IV^ afforded cationic species Ir^III^ and intermediate 69, which was deprotonated to give 65, while reaction of 69 with alcohols (R′OH) afforded the target product 64.

**Scheme 21:**
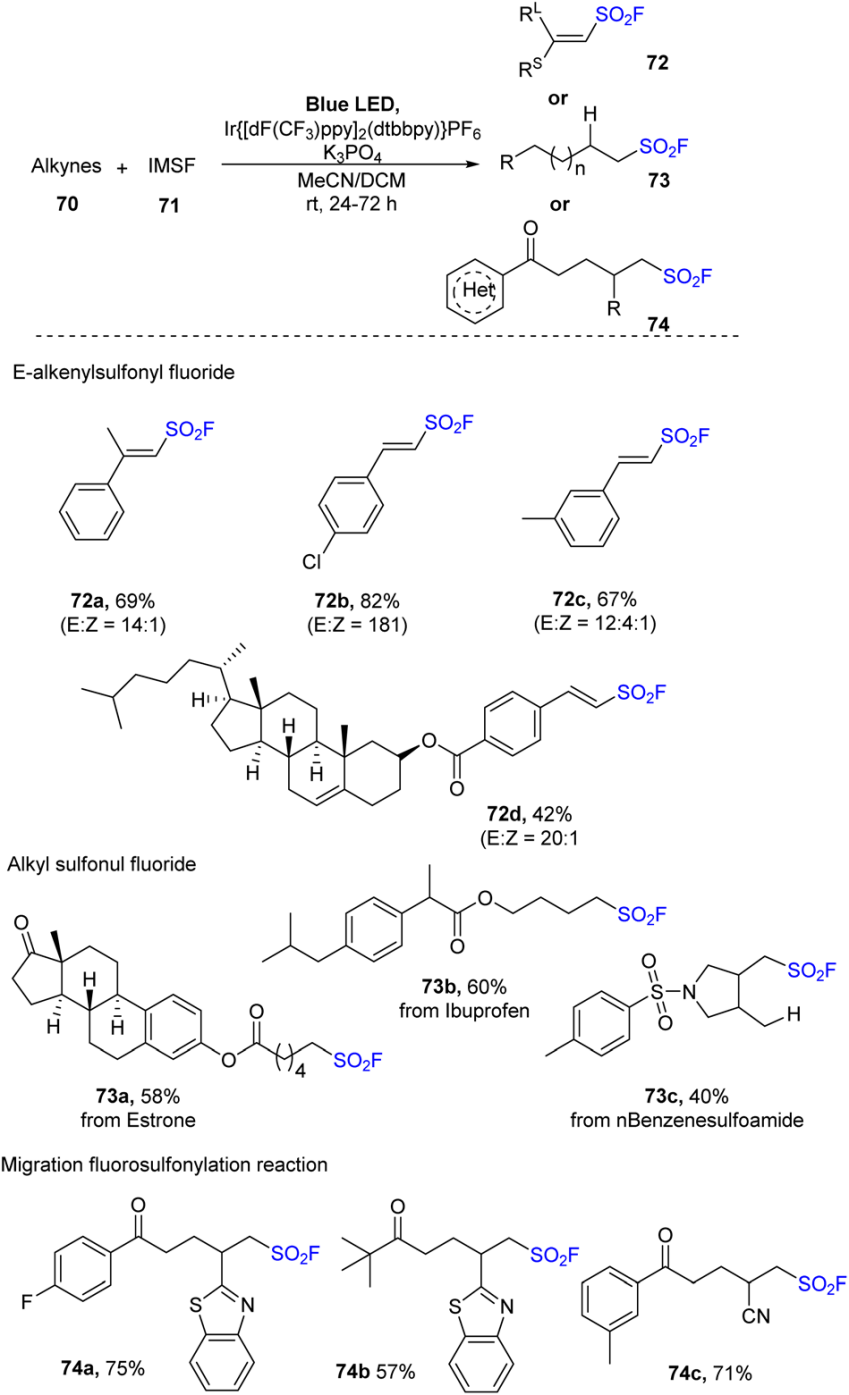
Radical alkenylsulfonyl fluoride reaction.

Wang and co-workers developed a fluorosulfonate cationic salt reagent with the goal of discovering efficient and stable reagents for generation of FSO_2_ radical, and they applied it for fluorosulfonylation of unsaturated hydrocarbons in 2022.^83^ In this study, benzimidazolium sulfonates salts (IMSF) were reacted with olefins as starting materials in the presence of 4CzIPN or Ir{[dF(CF_3_)ppy]_2_(dtbbpy)}PF_6_ as a photocatalyst and KH_2_PO_4_ under blue LED irradiation at room temperature (Scheme 21). Using the reaction, terminal alkene with di-substitutes was transformed into the corresponding product 72a in moderate yield with high stereoselectivity. Reactions using styrene derivatives with poor- and rich-electron rings afforded the desired products (72b, 72c) in 82–67% yields. Cholesterol derivative was readily converted to the target alkenyl sulfonyl fluoride 72d in moderate yield. Various substrates were successfully used in radical hydrofluorosulfonylation to generate alkyl sulfonyl fluorides. For example, derivatives of estrogen, ibuprofen, and benzenesulfonamide were well tolerated to afford the desired products (73a–73c) in 40–60% yields. This strategy was also applied for radical migration fluorosulfonylation using unsaturated tertiary alcohol as substrates. A variety of tertiary alcohols containing functional groups including (hetero)aryl groups, linear, or cyclic alkyl groups smoothly underwent this process to give the corresponding ketones (74a–74c) with good yields.

**Scheme 22:**
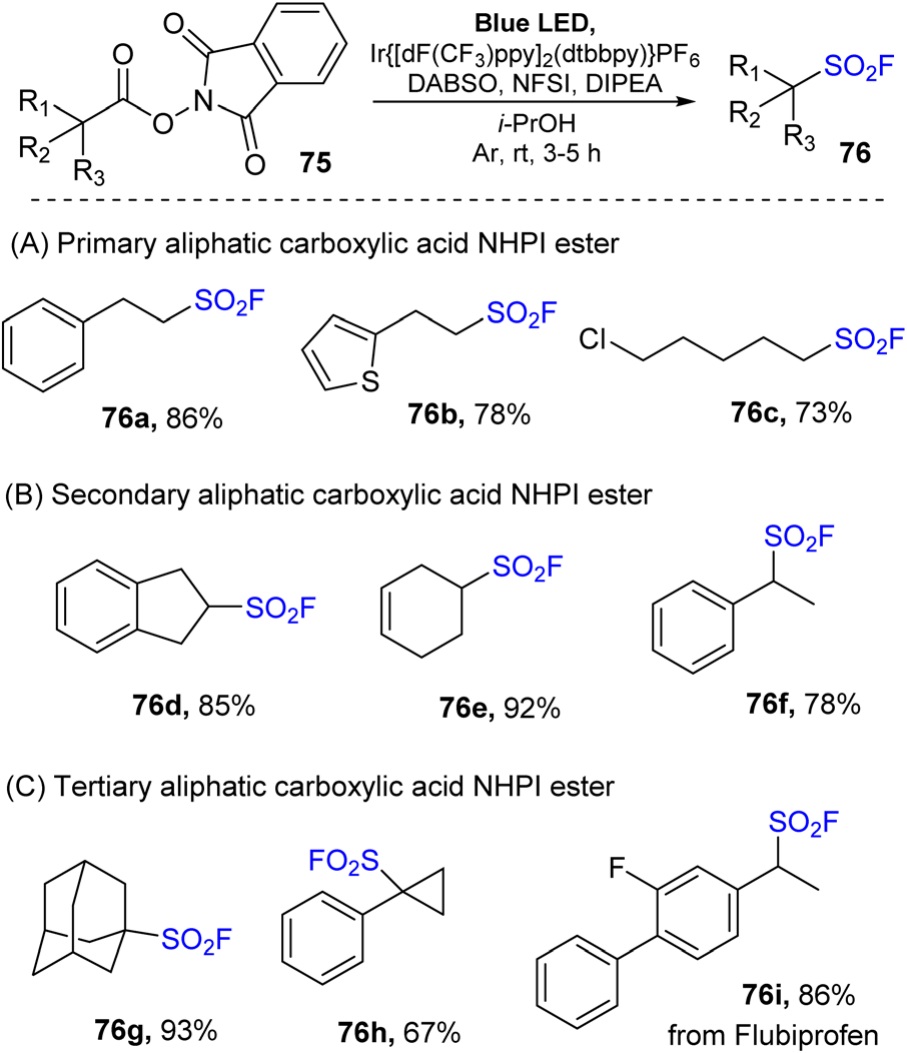
Synthesis of aliphatic sulfonyl fluorides *via* decarboxylative radical fluorosulfonylation.

Another study using redox-active *N*-hydroxyphthalimide (NHPI) esters of aliphatic carboxylic acid as precursors for synthesis of sulfonyl fluoride was reported by Nie and co-workers in 2022.^84^ Instead of direct utilization of SO_2_F from vinyl sulfonyl fluoride as in the previously reported method,^73^ multicomponent reactions between NPHI ester, DABSO, and NSFI in the presence of Ir[dF(CF_3_)ppy]_2_(dtbbpy)PF_6_ as a photocatalyst and DIPEA as a reductant under blue LED in *i*PrOHs at room temperature were carried out to give sulfonyl fluorides (Scheme 22). Various NHPI esters of carboxylic acids including primary, secondary, and tertiary derivatives were successfully employed for fluorosulfonylation. Primary carboxylic acids bearing different functional groups such as phenyl, thienyl, and chloride groups were well tolerated with the reaction to give the corresponding products (76a–76c). Secondary acids with dihydro-indene, cyclohexene, and α-methyl benzyl derivatives were readily transformed into the desired products (76d–76f) in high yields. One benefit of this approach is the direct conversion of tertiary substrates to the corresponding alkyl sulfonyl fluorides (76g, 76h) with high yields. Notably, fluorosulfonylation of drug was successfully achieved to give the target product 76i under mild conditions.

**Scheme 23:**
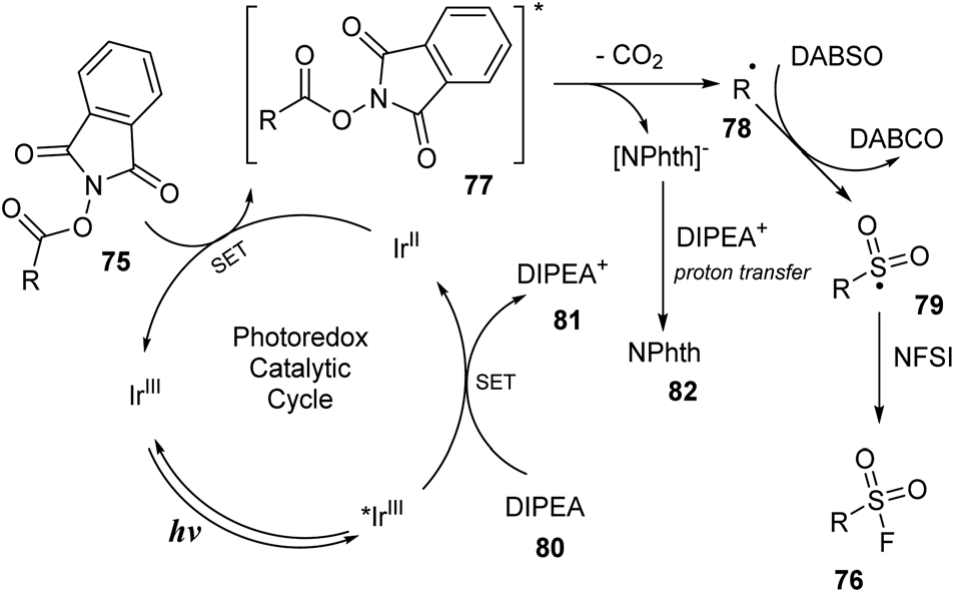
Passible mechanism of visible light-mediated decarboxylative radical fluorosulfonylation.

A proposed mechanism of this reaction is shown in Scheme 23. Blue LED excited photocatalyst Ir^III^ to active *Ir^III^, which was reduced by DIPEA 80 to form DIPEA˙^+^ cation radical 81 and Ir^II^. Then, Ir^II^ underwent the single-electron transfer (SET) process with NHPI ester 75 to generate NHPI˙^−^ ester radical 77 and Ir^III^. The radical 77 was self-decomposed to provide alkyl radical 78, CO_2_ gas, and NPhth^−^ anion. The NPhth^**−**^ anion underwent a proton transfer process with DIPEA˙^+^81 to give NPhth 82. The insertion of SO_2_ into 78 by reaction with DABSO followed by introduction of the F atom from NSFI led to final product 76.

**Scheme 24:**
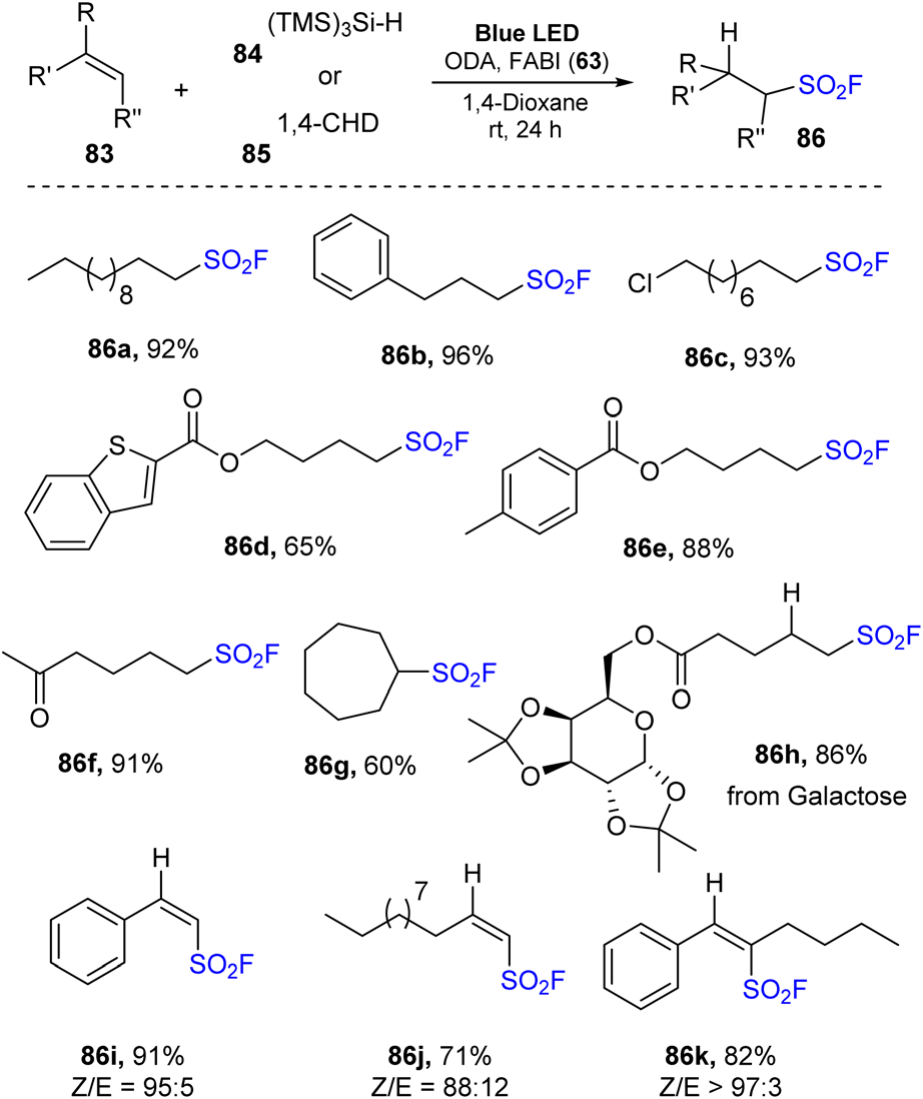
Radical hydro-fluorosulfonylation of alkenes.

In 2022, Liao and co-workers employed benzimidazolium fluorosulfonate cationic salt to generate fluorosulfonyl radical precursors in radical hydro-fluorosulfonylation of unactivated alkenes (Scheme 24).^85^ Alkyl sulfonyl fluoride products were obtained *via* reaction of alkenes with 1-fluorosulfonyl 2-aryl benzoimidazolium (FABI) triflates in the presence of cyclohexa-1,4-hexadiene (or TMS_3_SiH) as a hydrogen donor and oxygen-doped anthanthrene (ODA) as a photocatalyst in 1,4-dioxane under irradiation of blue LEDs at room temperature for 24 hours. Using this method, both linear and cyclic unactivated alkenes were transformed into the corresponding products with high yields. Alkenes containing functional groups including halides, esters, heteroaryl, tosylate, and ketones were tolerated with this method, generating the target products (86a–86f) in 65–96% yield. Moreover, the hydrofluoro-sulfonylation process employing cyclic olefin was easily carried out to produce the corresponding sulfonyl fluoride (86g). Diverse derivatives of natural products, peptides, and drugs were subjected to the standard reaction conditions to afford the corresponding product 86h in good yield, suggesting that this method has potential in chemical biology and drug discovery. The scope of the protocol was extended to radical hydro-fluorosulfonylation of alkynes to synthesize valuable alkenyl sulfonyl fluorides. Terminal alkynes with various groups such as (hetero)aryl alkynes or alkyl alkynes were readily tolerated with this method to provide the corresponding alkenyl sulfonyl fluorides (86i, 86j). Reaction of internal alkyne produced the product (86k) in 82% with high *Z*-selectivity (*Z*/*E* = 97 : 3).

**Scheme 25:**
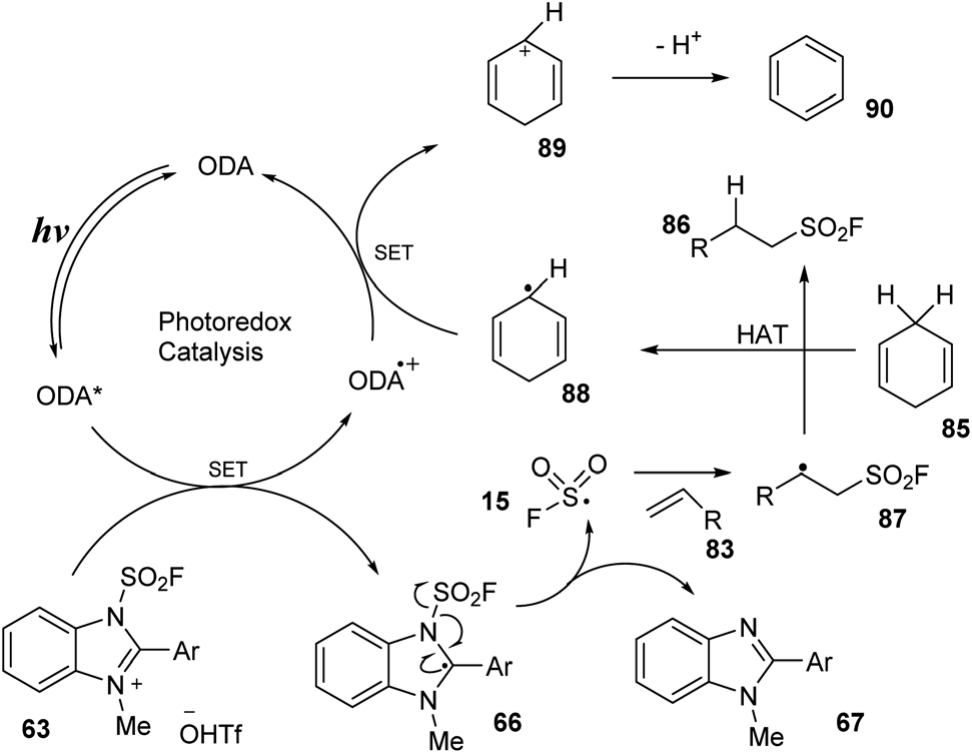
Proposed mechanism for radical hydro-fluorosulfonylation of alkenes.

A proposed mechanism of this radical hydro-fluorosulfonylation is shown in Scheme 25. Blue LED light excited photocatalyst ODA to the ODA* state, which then underwent SET with benzimidazolium fluorosulfonate salt 63 to form intermediate 66 and ODA˙^+^. Intermediate 66 was rapidly decomposed to FSO_2_ radical 15 and compound 67. The FSO_2_ radical 15 reacted with alkene substrate 83 to generate intermediate 87, which underwent hydrogen atom transfer with cyclohexa-1,4-diene (CHD) 85 to provide desired product 86 and radical 88. Another SET process was carried out between radical 88 and ODA˙^+^ to give cation CHD^+^89 and returned ground state catalyst ODA. Cation CHD^+^89 released one proton to form benzene.

**Scheme 26:**
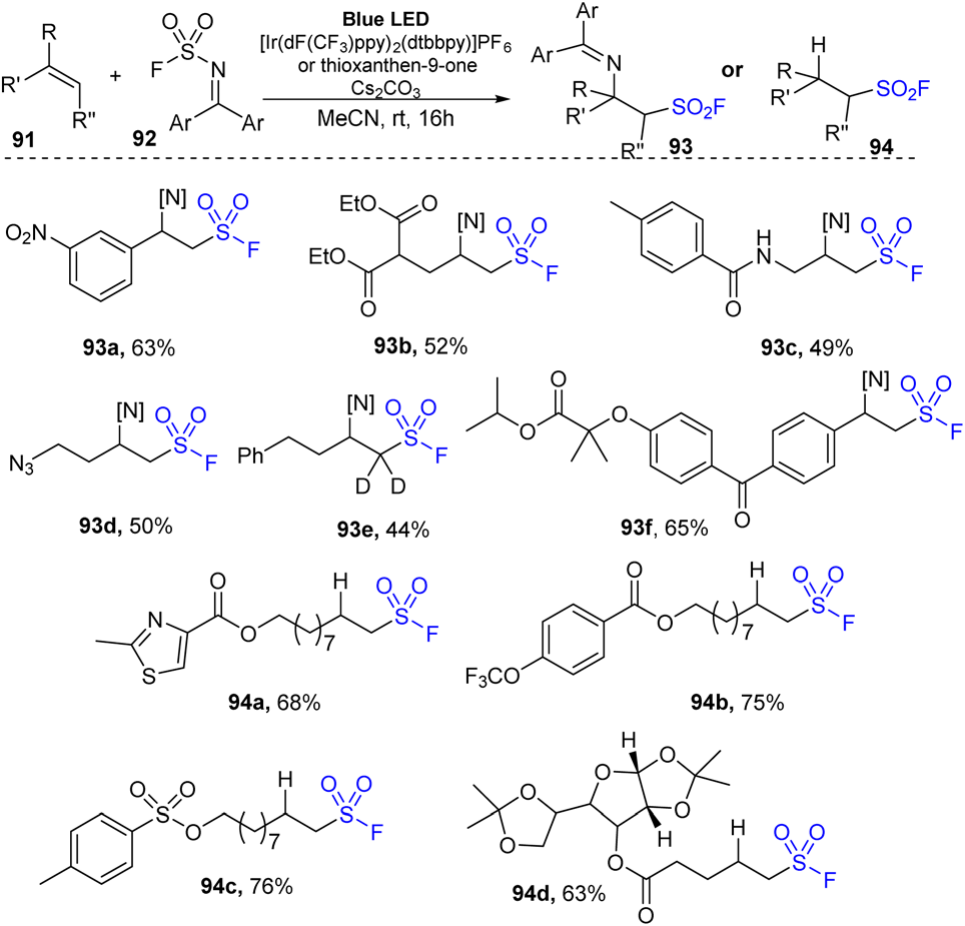
Imino-fluorosulfonylation and hydro-fluorosulfonylation from alkenes.

Recently, Glorious and co-workers developed a novel method to synthesize β-amino sulfonyl fluorides through addition of both amine and SO_2_F groups into alkenes using (diphenylmethylene)sulfamoyl fluoride as a novel reagent for imino-fluorosulfonylation (Scheme 26).^86^ Reactions were carried out in a single step by treatment of alkenes with (diphenylmethylene)sulfamoyl fluoride in the presence of thioxanthone as a photocatalyst in DMC under blue LED irradiation at room temperature for 24 hours. Styrenes containing various functional groups were tolerated with this process to give the corresponding di-substitutes product 93a in good yield. Using the method, terminal alkenes with malonate diester and amine groups were readily transformed into sulfonyl fluorides (93b, 93c). Straight chain alkene bearing azide group was also tested and afforded the corresponding product 93d in 50% yield. Reaction of deuterium-alkene also generated the sulfonyl fluoride product 93e in moderate yield. Radical hydro-fluorosulfonylation reactions were conducted with modified reaction conditions using [Ir(dF(CF_3_)ppy)_2_(dtbbpy)]PF_6_ as a photocatalyst and 2,4,6-triisopropylbenzenethiol (TripSH) as a hydrogen atom donor. In the process, straight-chain alkenes bearing heteroarene, sulfonate ester, and trifluoromethoxy groups were smoothly transformed into the desired alkyl sulfonyl fluorides (94a–94c) in 68–76% yields. Some alkenes derived from natural products or drugs were successfully used in this method, giving the corresponding products with good yield (94d).

**Scheme 27:**
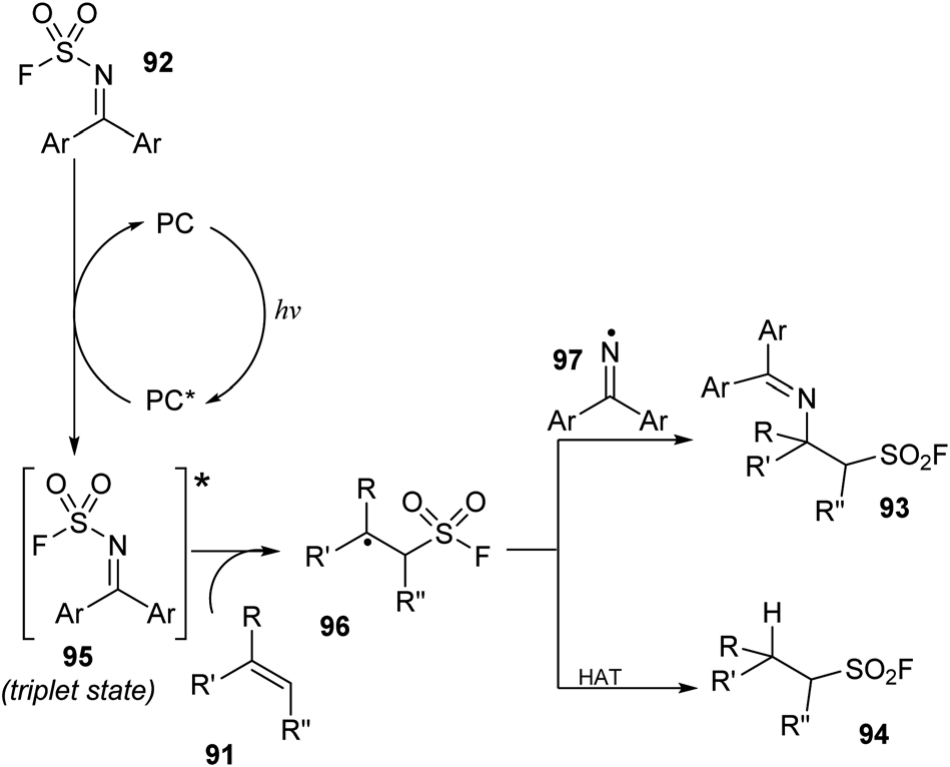
A plausible reaction mechanism for imino-fluorosulfonylation and hydro-fluorosulfonylation from alkenes.

A proposed mechanism of this method is illustrated in Scheme 27. Photocatalyst PC was excited by irradiation of blue LED light and interacted with substrate 92 ((diphenylmethylene)sulfamoyl fluoride) to form triplet state intermediate 95 and regenerated the photocatalyst in the ground state. The N–S bond of intermediate 95 was broken to provide sulfonyl fluoride radical and iminyl radical 97. The sulfonyl fluoride radical reacted with alkene 91 to generate alkyl sulfonyl fluoride radical 96, which further reacted with iminyl radical 97 to generate β-amino sulfonyl fluoride 93. In another pathway, the alkyl sulfonyl fluoride radical received a hydrogen atom from a donor to give aliphatic sulfonyl fluoride product 94.

### 2.2. Visible light induced synthesis of sulfonic esters (S–O bond formation)

Sulfonic esters have been considered an important structure for many organic compounds and pharmaceuticals. Several non-visible-light-mediated reactions have been reported for synthesis of these compounds. However, studies of visible-light-mediated reactions have not widely been reported.

**Scheme 28:**
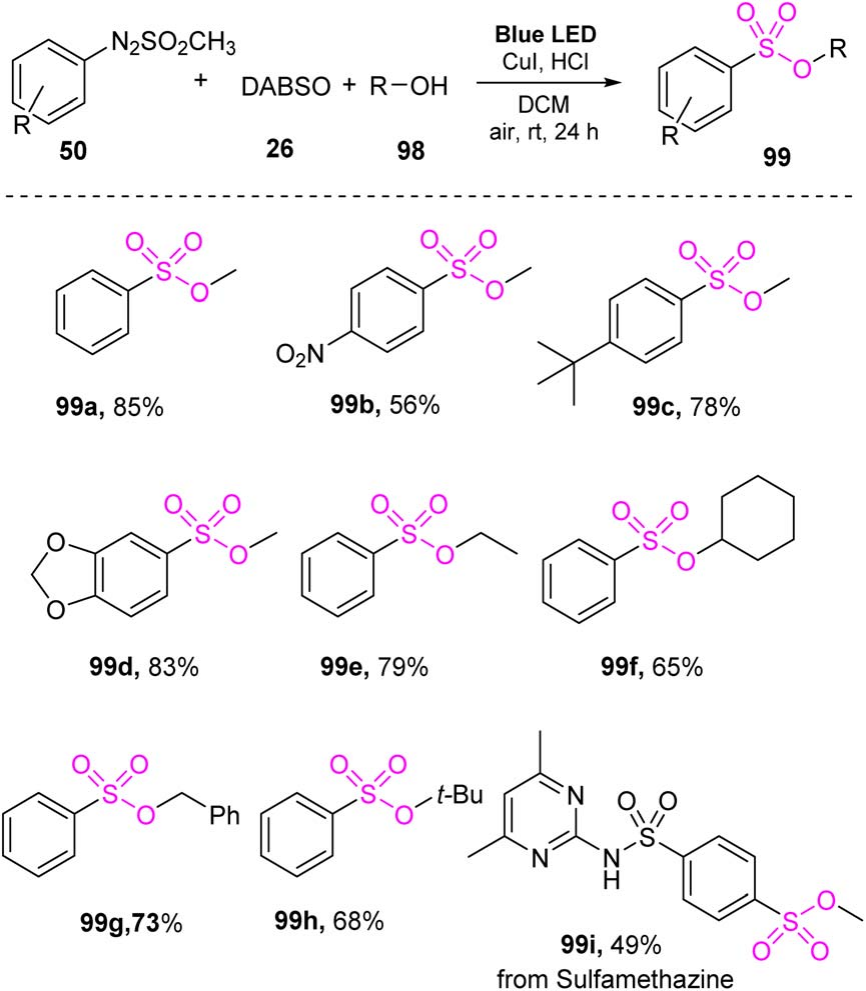
Visible-light-induced synthesis of sulfonate esters from arylazo sulfones.

In 2022, Kim and co-workers developed a visible-light-mediated synthetic method for producing sulfonic esters from arylazo sulfones without a photocatalyst.^87^ Reaction of arylazo sulfone salts with DABSO and alcohols was conducted through a one-pot reaction in CH_2_Cl_2_ under visible-light irradiation at room temperature. Copper salt as a catalyst and a small amount of hydrochloric acid as an additive were employed to achieve the synthesis of sulfonic esters (Scheme 28). A wide range of arylazo sulfone derivatives bearing various substituents including electron-withdrawing and electron-donating groups was well tolerated in the reactions to give the corresponding products (99a, 99b, and 99c) with high yields. Arylazo sulfones bearing two substituents and a heterocyclic group underwent this process to afford the product 99d with 83% yield. Notably, the sulfamethazine moiety (used in drugs) was converted to sulfonic ester product 99i with 49% yield. Various alcohols have been tested for this method, and straight chain alcohols, cyclic alcohols, and benzylic alcohols provided the desired products (99e–99h) with moderate yields.

**Scheme 29:**
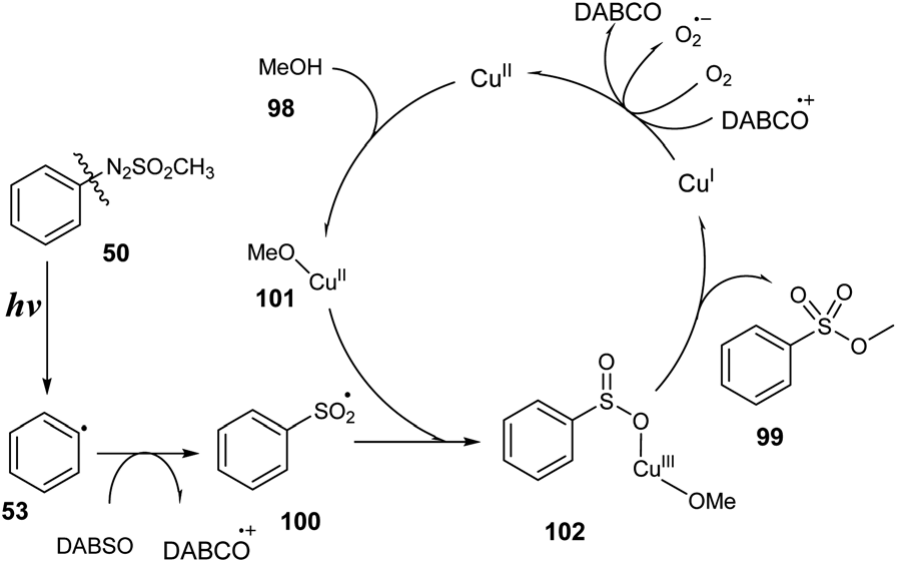
Proposed reaction mechanism for visible-light-induced synthesis of sulfonic esters.

A plausible mechanism was proposed as shown in Scheme 29. Arylazo sulfone salt 50 was decomposed under visible light irradiation to provide aryl radical 53, methyl sulfonyl radical, and N_2_ gas. Aryl radical 53 was reacted with DABSO to generate aryl sulfonyl radical 100, which interacted with the complex of copper salt and methanol to provide intermediate 102. In the last step, intermediate 102 self-decomposed to form the final product 99 and copper I, which was oxidized by O_2_ in air or DABCO cation to return copper II.

### 2.3. Visible light induced synthesis of sulfonamides (S–N bond formation)

Sulfonamide compounds have been recognized as compounds with valuable biochemical properties, and they have been used in many fields, including drugs. Since photochemical reactions for the synthesis of sulfonamides were first reported in 2016, a series of photochemical reaction methods has been used to prepare sulfonamides.

**Scheme 30:**
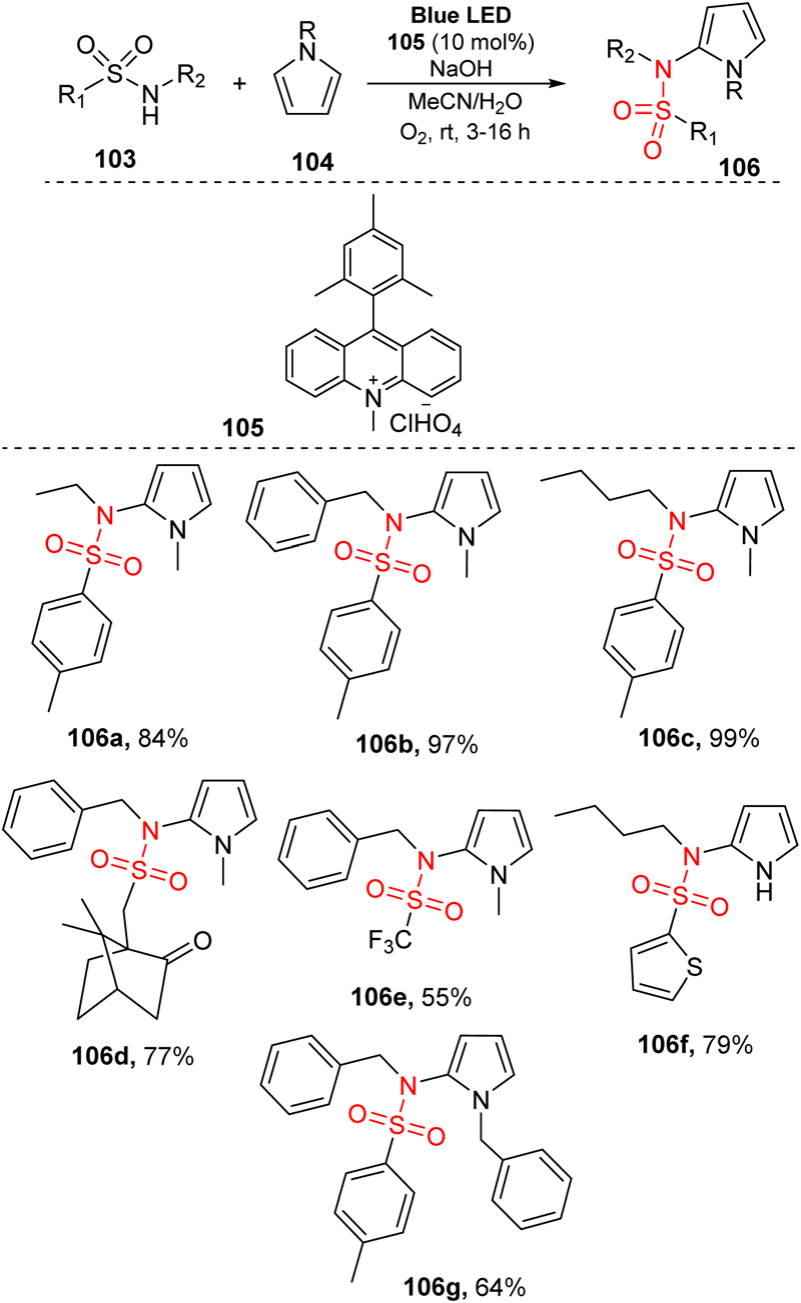
Synthesis of *N*-(2-pyrrole)-sulfonamides from sulfonamides and pyrroles.

In 2016, König and co-workers reported metal-free photoreaction C–H sulfonamidation of pyrroles under blue LED irradiation.^88^ In their study, sulfonamides were reacted with *N*-substituted pyrroles in the presence of 9-mesityl-10-methylacridinium perchlorate as a photocatalyst, NaOH, and O_2_ gas in a mixture of MeCN and H_2_O under irradiation of visible light at room temperature for 3 to 16 hours (Scheme 30). A series of sulfonamides containing various substitutes including *N*-alkyl and *N*-benzyl groups as well as *S*-alkyl and *S*-(hetero)aryl groups was readily reacted with N–Me–pyrrole to generate the corresponding products (106a–106d) in good yields. Reaction using a substrate with a benzyl-trifluoro sulfonamide group generated the desired product 106e in moderate yield. A variety of pyrrole derivatives such as *N*-benzylpyrrole was well accommodated under the reaction conditions, affording the target sulfonamide products (106f–106g) with good yields.

**Scheme 31:**
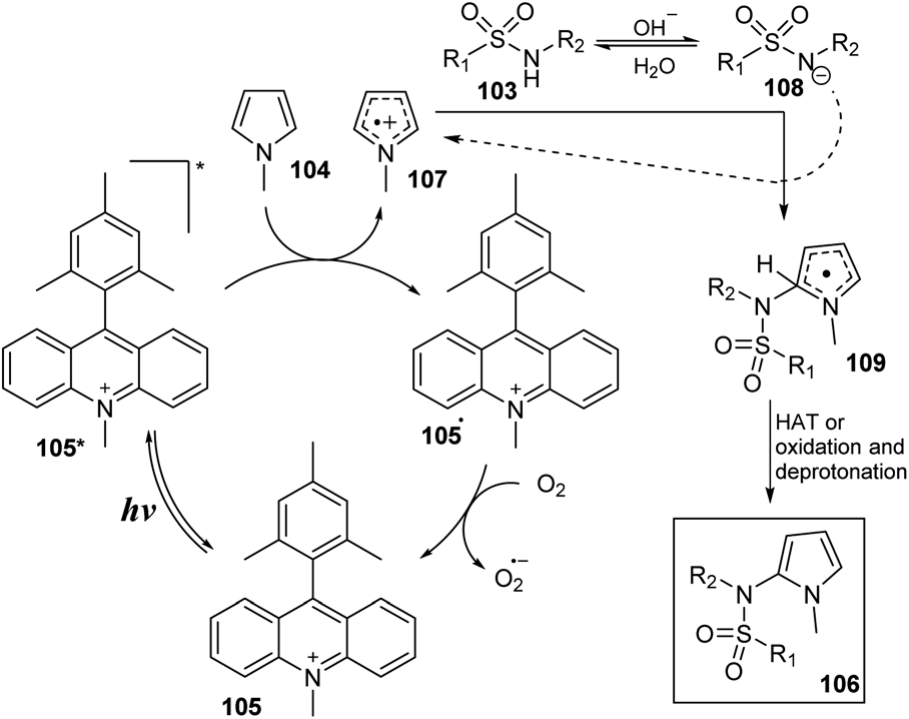
Proposed catalytic cycle for synthesis of *N*-(2-pyrrole)-sulfonamides from sulfonamides and pyrroles.

A mechanism was proposed as shown in Scheme 31. Under irradiation of blue LED, photocatalyst A was transferred to excited state 105*. Compound 105* was readily reduced by *N*-methylpyrrole 104 to form photocatalyst radical 105˙ and radical cation 107. In a basic environment, sulfonamide 103 removed one proton to give anion 108, which then reacted with radical cation 107 to generate radical 109. This radical underwent the HAT process to provide the desired product 106. The photocatalyst radical 105˙ reacted with O_2_ to regenerate photocatalyst 105 at ground state and anion radical O_2_˙^−^.

**Scheme 32:**
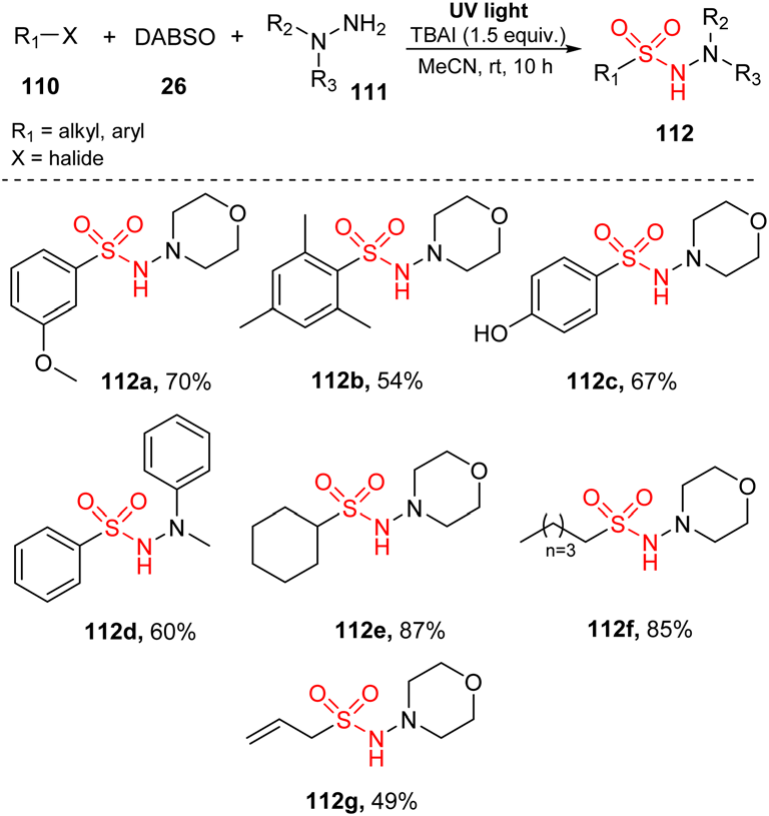
Photo-induced aminosulfonylation of aryl/alkyl halides, sulfur dioxide, and hydrazines.

In 2016, Wu and co-workers reported a visible-light-induced reaction to synthesize *N*-aminosulfonamides through insertion of SO_2_ into organic molecules.^89^ This reaction was achieved *via* tricomponent reactions using aryl/alkyl halides, hydrazines as substrates, and DABSO in the presence of TBAI in MeCN under irradiation of a mercury lamp at room temperature for 10 hours (Scheme 32). This method tolerated all aryl bromide substrates with various substitutes including electron-withdrawing groups, electron-donating groups, and halide groups, regardless of position and number of substituents, to give the corresponding products (112a, 112b). Reaction of a substrate containing 4-hydroxy group provided the desired product 112c in 67% yield. A variety of hydrazines was employed for this aminosulfonylation to produce the corresponding product 112d in good yields. Additionally, desired products bearing alkyl groups such as cyclic alkyl 112e, linear alkyl 112f, and double bond 112g were smoothly generated in moderate yields.

**Scheme 33:**
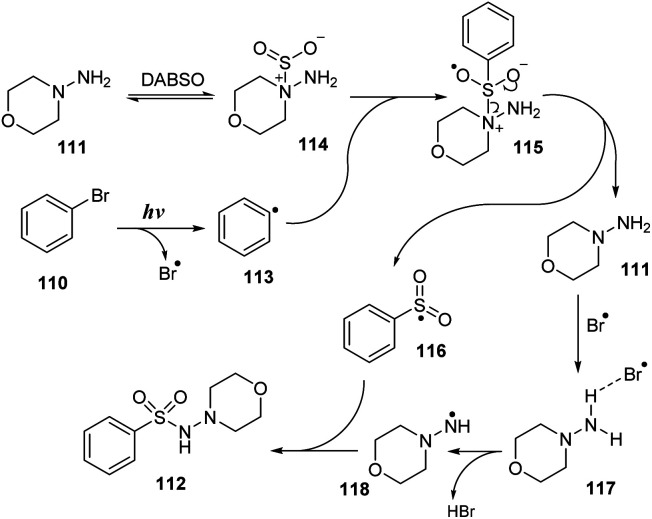
A plausible mechanism for photo-induced aminosulfonylation of aryl/alkyl halides, sulfur dioxide, and hydrazines.

A plausible mechanism was proposed as shown in Scheme 33. Interaction of hydrazine 111 with DABSO created the hydrazine–SO_2_ complex 114. At the same time, bromobenzene 110 underwent homologous cleavage of the C–Br bond under ultraviolet irradiation to give phenyl radical 113 and Br radical. Phenyl radical 113 then reacted with complex 114 to form intermediate 115, which was readily decomposed to provide phenyl sulfonyl radical 116 and regenerated hydrazine 111. Hydrazine 111 further reacted with Br radical to give intermediate 117, and removal of HBr formed radical 118. This radical reacted with phenyl sulfonyl radical 116 to provide the desired product 112.

**Scheme 34:**
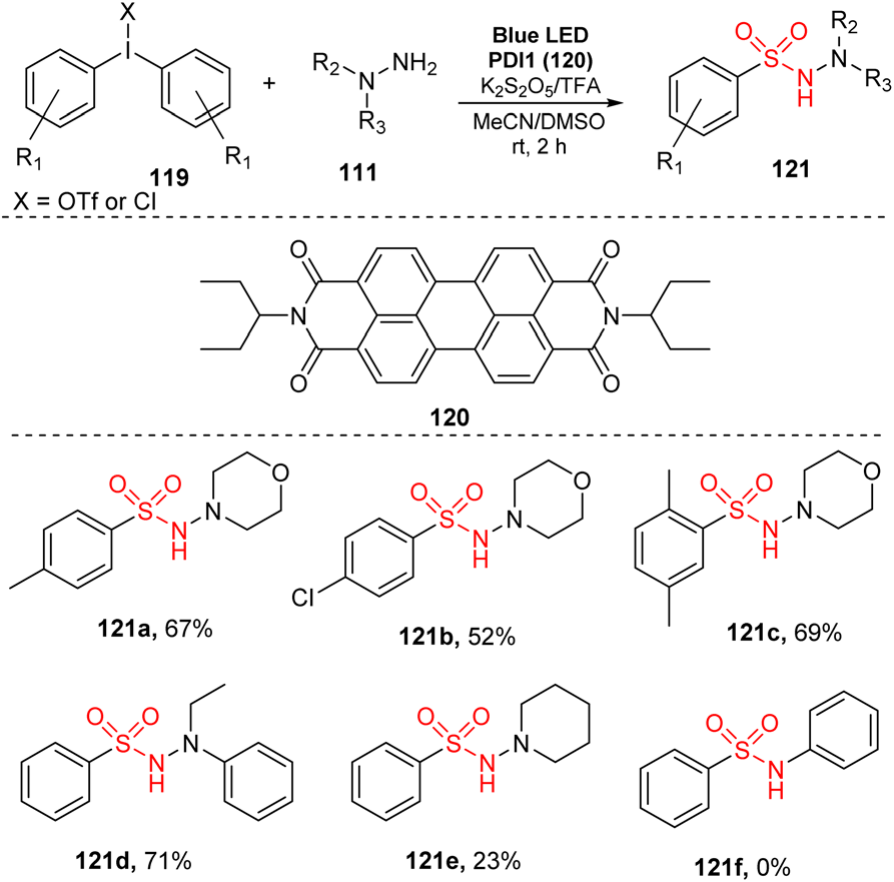
Synthesis of *N*-aminosulfonamides using diaryliodonium salts, hydrazines and sulfur dioxide.

In 2017, Manolikakes and co-workers reported a three-component reaction of diaryliodonium salts, hydrazines, and K_2_S_2_O_5_ to produce *N*-aminosulfonamides (Scheme 34).^90^ The reactions were conducted in the presence of perylenediimide as a photocatalyst and TFA in a mixture of MeCN and DMSO under irradiation of a blue LED at room temperature for 2 hours. Reaction of diaryliodonium salts bearing various functional groups on aryl rings provided the corresponding aminosulfonamides (121a, 121b) in moderate yields. Notably, changing the position and number of substituents had little effect on the reaction efficiency. For example, product 112c with 2,5-dimethyl group was produced with 69% yield under standard reaction conditions. When hydrazines were used as substrates, the reaction gave the target aminosulfonylated product 121d with good yield, while reaction of *N*-aminopiperidine afforded the corresponding product 121e with only 23% yield. Aniline was not tolerated in this method.

**Scheme 35:**
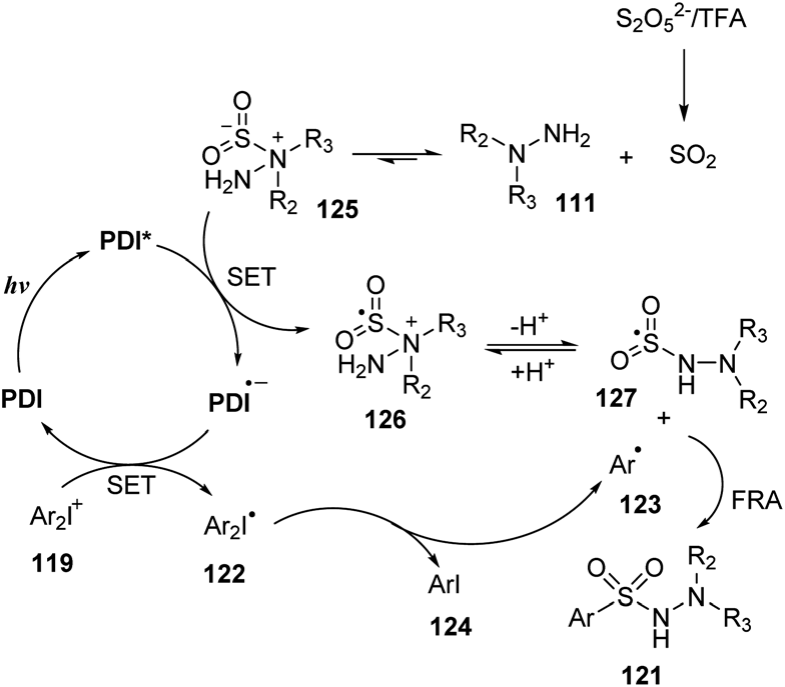
Proposed mechanism of catalytic photoredox pathway for synthesis of *N*-aminosulfonamides using diaryliodonium salts.

A plausible mechanism for the aminosulfonylation proposed by Manolikakes and co-workers is shown in Scheme 35. Sulfur dioxide SO_2_, which was formed by reaction of bisulfite salt K_2_S_2_O_5_ and TFA, interacted with hydrazine 111 to generate the stable hydrazine-sulfur dioxide 125. At the same time, catalyst PDI was excited by blue LED light and was transformed to photoexcited PDI*, which underwent SET with compound 125 to provide radical cation 126 and reduced catalyst PDI˙^−^. Removal of radical cation 126 formed sulfonyl radical 127. The reduced catalyst PDI˙^−^ underwent another SET process with diaryliodonium salt 119 to give catalyst PDI and radical 122, which further decomposed to form aryl iodine 124 and aryl radical 123. This radical combined with sulfonyl radical 127 to generate final product 121.

**Scheme 36:**
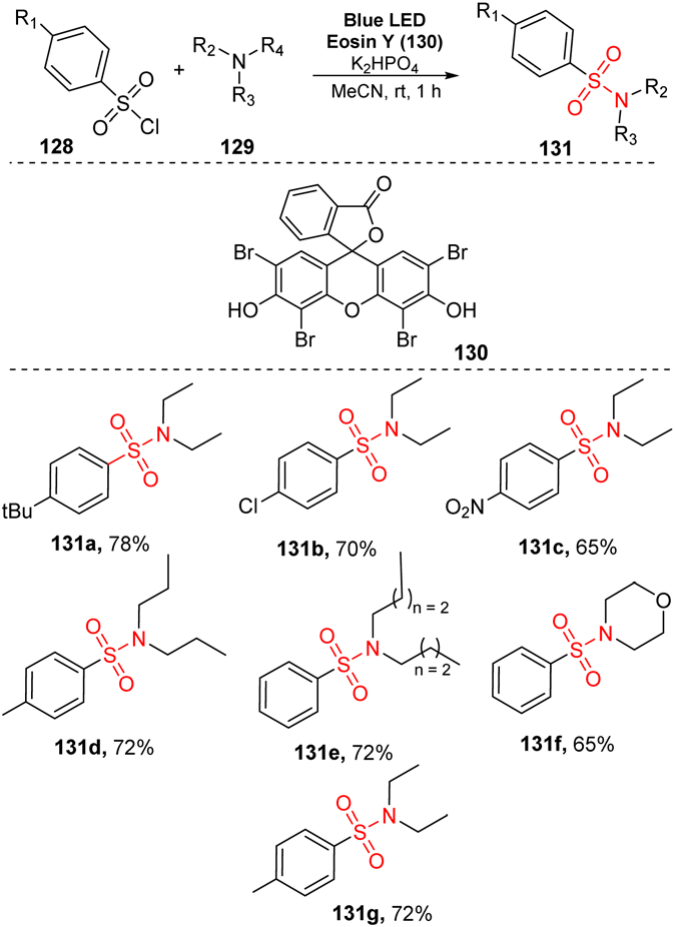
Synthesis of sulfonamides *via* reaction of aliphatic amines with arenesulfonyl chlorides in the presence of eosin Y.

In 2017, Zhang and co-workers developed an aerobic oxidative reaction of trialkylamines with arenesulfonyl chlorides to produce sulfonamides without transition-metal catalyst.^91^ In that study, aliphatic amines reacted with arenesulfonyl chlorides in the presence of eosin Y as a photocatalyst and K_2_SO_4_ as a base in MeCN under irradiation of visible light at room temperature for 1 hour (Scheme 36). Sulfonyl chlorides with electron-donating groups, electron-withdrawing groups, and halide groups were well tolerated in this process, providing the corresponding sulfonamides (131a–131c) in 65–78% yields. Using the process, symmetrical tertiary amines with three alkyl groups (*e.g.*, tripropylamine and tributylamine) were smoothly converted to the desired products (131d, 131e) in good yields. When asymmetric tertiary amines such as *N*-ethylmorpholine and diethyl(methyl)amine were employed, the target sulfonamides (131f, 131g) were obtained in 65–72% yields.

**Scheme 37:**
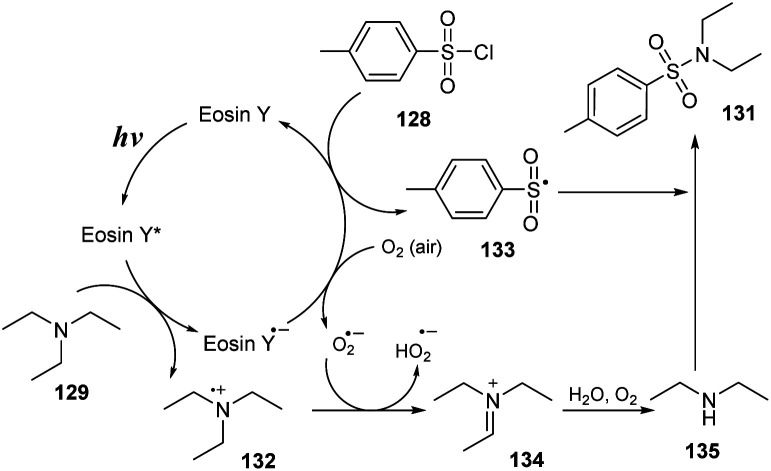
Proposed mechanism for synthesis of sulfonamides *via* reaction of aliphatic amines with arenesulfonyl chlorides.

A proposed mechanism for this method is described in Scheme 37. Photocatalyst eosin Y (EY) was transferred to the excited state EY* under irradiation with a blue LED. It was then reduced by triethylamine 129 to generate EY˙^−^ and a radical cation 132. EY˙^−^ then underwent SET with O_2_ (or 128) to regenerate EY and form O_2_˙^−^ (or sulfonyl radical 133). At the same time, 128 reacted with O_2_ to provide O_2_˙^−^ and radical 133. O_2_˙^−^ captured one proton from radical cation 132 to give HO_2_˙^−^ and iminium cation 134. Cation 134 was hydrolyzed by H_2_O and O_2_ to produce amine 135, which further reacted with sulfonyl radical 133 to provide the final product 131.

**Scheme 38:**
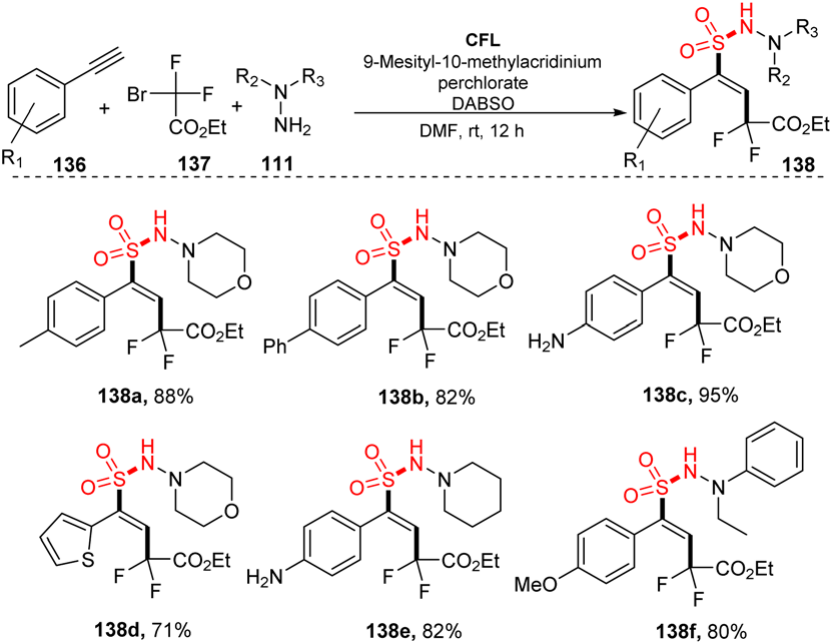
Visible-light-induced vicinal difluoroalkylation and aminosulfonylation of alkynes.

In 2017, Wu and co-workers reported vicinal difluoroalkylation and aminosulfonylation of alkynes *via* a four-component reaction.^92^ Alkynes, ethyl 2-bromo-2,2-difluoroacetate, hydrazines as substrates, and DABCO·(SO_2_)_2_ were used for the multicomponent reaction in the presence of 9-mes-10-methyl acridinium perchlorate as a photocatalyst in DMF under irradiation of a compact fluorescent light (CFL) at room temperature for 12 hours (Scheme 38). Using the process, aryl alkynes bearing substituents on the benzene ring (such as electron-donating groups, electron-withdrawing groups, and phenyl groups) were well transformed to the desired products (138a, 138b) in 82–88% yields. The process tolerated substrates bearing sensitive functional groups, and amino-substituted product 138c also was generated in high yield under the standard reaction condition. Heteroaryl-substituted substrate was tolerated with this process to provide product 138d in 71% yield. Reactions using hydrazines were smoothly carried out to form the corresponding products (135e, 138f).

**Scheme 39:**
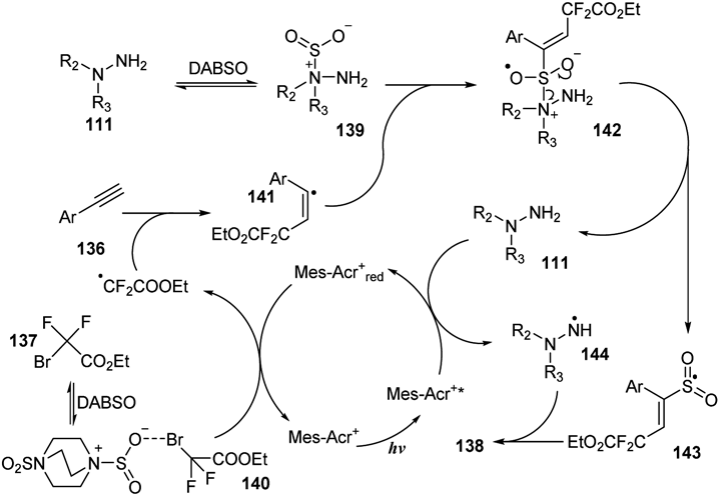
A plausible mechanism for visible-light-induced vicinal difluoroalkylation and aminosulfonylation of alkynes.

A proposed mechanism for this method is described in Scheme 39. Mes-Acr^+^ was excited by irradiation of blue LED light and was transferred to the Mes-Acr^+*^, which reacted with hydrazine 111 to afford radical 114 and Mes-Acr^+^_red_. At the same time, ethyl 2-bromo-2,2-difluoroacetate 137 combined with DABSO to form complex 140, which further interacted with Mes-Acr^+^_red_ to form a difluoroalkyl radical and the Mes-Acr^+^ ground state. The difluoroalkyl radical reacted with phenyl alkyne 136 to give alkenyl radical 141. Hydrazine 111 would also react with DABSO to provide complex 139, which combined with alkenyl radical 141 to give intermediate 142 that easily decomposed to produce radical 143 and regenerate hydrazine 111. Intermediate 143 interacted with radical 144 to afford the final product 138.

**Scheme 40:**
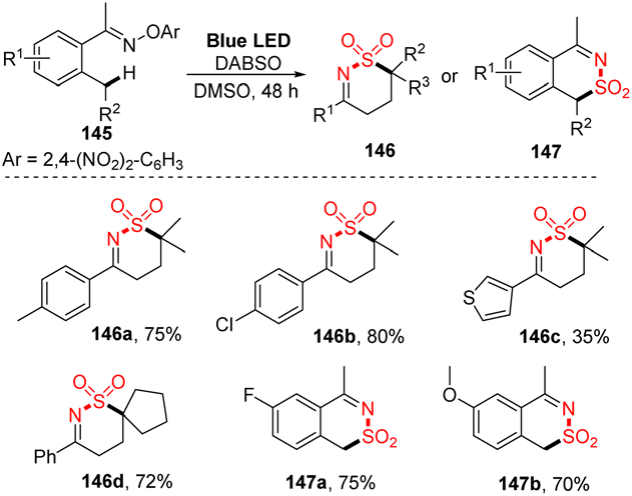
Visible-light-induced aminosulfonylation of an unactivated C(sp^3^)–H bond with sulfur dioxide.

Aminosulfonylation of unactivated C(sp^3^)–H bonds without any metals or photocatalyst was developed by Wu and co-workers in 2017.^93^ To carry out the photoreaction for insertion of SO_2_, *O*-aryl oxime derivatives were treated with DABSO in DMSO under irradiation of blue LED light at room temperature for 48 hours (Scheme 40). Using the protocol, a variety of *O*-aryl oximes was successfully transformed into the corresponding 1,2-thiazine 1,1-dioxides in good yields. Substrates with functional groups such as electron-withdrawing groups and electron-donating groups as well as heterocyclic groups were tolerated with the reaction to provide the desired products (146a–146c) in 35–80% yield. When substrates with a cycloalkyl group around the C(sp^3^)–H bond were used, the desired product (146d) was smoothly obtained in good yield. Reaction of substrates containing a benzylic carbon afforded various 1*H*-benzo[*d*][1,2]thiazine 2,2-dioxide products (147a, 147b) in good yields.

**Scheme 41:**
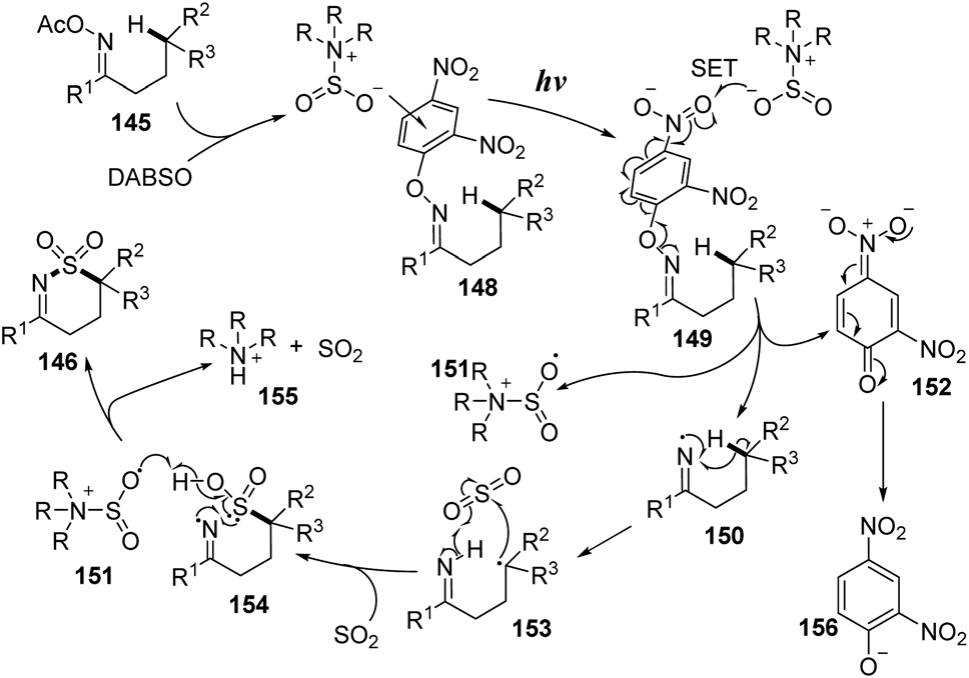
Plausible mechanism for visible-light-induced aminosulfonylation of an unactivated C(sp^3^)–H bond with sulfur dioxide.

A proposed mechanism of this method is illustrated in Scheme 41. The *O*-2,4-dinitrophenyl oxime 145 was reacted with DABSO to form photosensitive complex 148, which was excited by visible-light irradiation and underwent intramolecular SET. After cleavage of the N–O bonds, iminyl radical 150, sulfonyl amide radical 151, and intermediate 152 were formed. The iminyl radical 150 further participated in 1,5-H atom migration to give radical 153. Next, sulfur dioxide was inserted into radical 153 to yield intermediate 154, which interacted with sulfonyl amide radical 151 to generate the desired product 146.

**Scheme 42:**
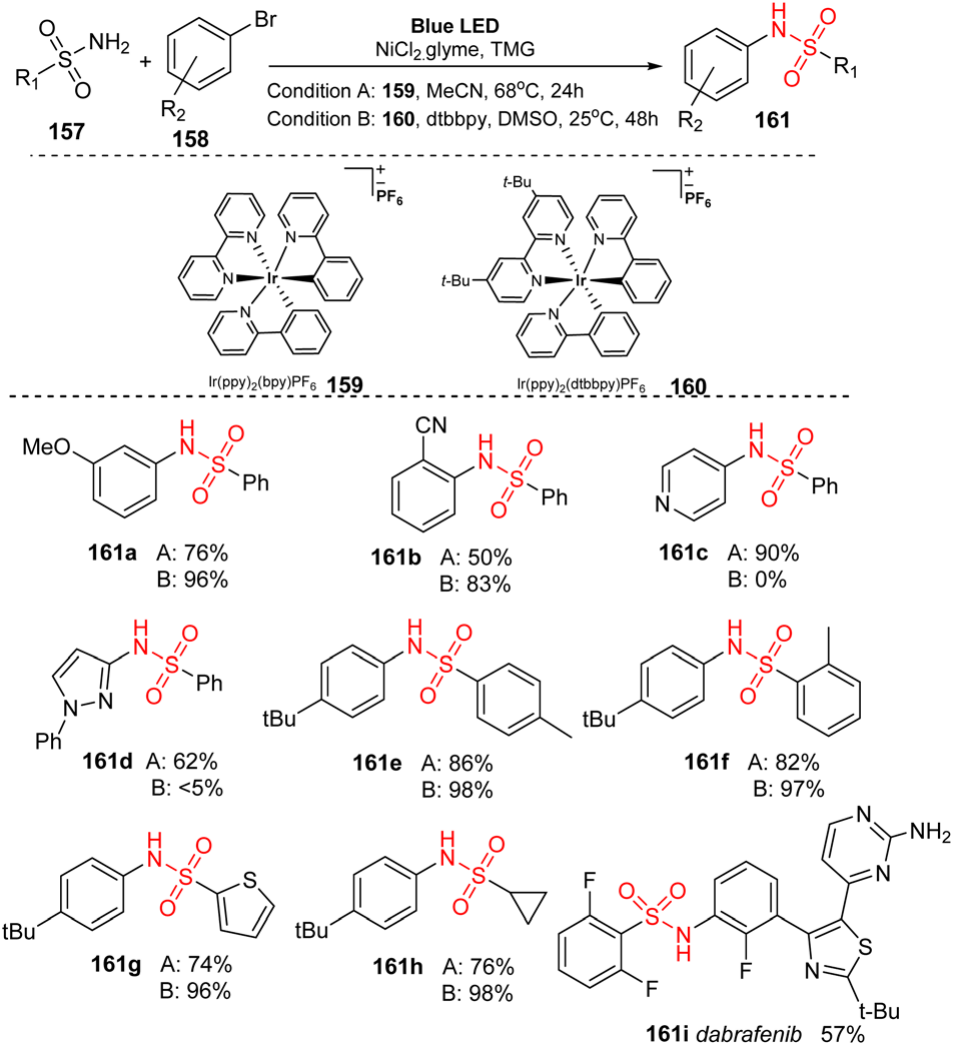
Synthesis of sulfonamides *via* photosensitized nickel-catalyzed cross-coupling.

In 2017, MacMillan and co-workers developed a nickel/iridium-catalyzed reaction to form C–N bonds for sulfonamidations.^94^ In this study, sulfonyl amides were reacted with (hetero)aryl bromide in the presence of Ir(ppy)_2_(bpy)PF_6_ and NiCl_2_·glyme as catalysts and tetramethylguanadine (TMG) as a base under irradiation of a blue LED (Scheme 42). In ligand-free conditions (condition A), the reactions were conducted in MeCN at 68 °C for 24 hours. In the presence of a ligand (condition B), 4,4-di-*tert*-butyl-2,2-dipyridyl (dtbbpy) was added to the mixture, and reactions were carried out in DMSO at 25 °C for 48 hours. Most aryl halides bearing electron-donating and electron-withdrawing groups were well tolerated in the reactions, providing the corresponding products (161a, 161b) with good yields. However, reaction yields from condition A were often lower than those from condition B. In contrast, heteroaryl halide substrates including pyridine halide or 5-membered ring heterocyclic halide were readily converted to the corresponding products (161c, 161d) in higher yields without addition of dtbbpy. Additionally, various (hetero)aryl sulfonamides were successfully employed for this process, generating the corresponding products (161e–161g). Reaction of alkyl sulfonamides also smoothly provided the desired product 161h in high yield. Dabrafenib 161i, a selective B-Raf kinase inhibitor, was successfully synthesized by this protocol with 57% yield in a single step.

**Scheme 43:**
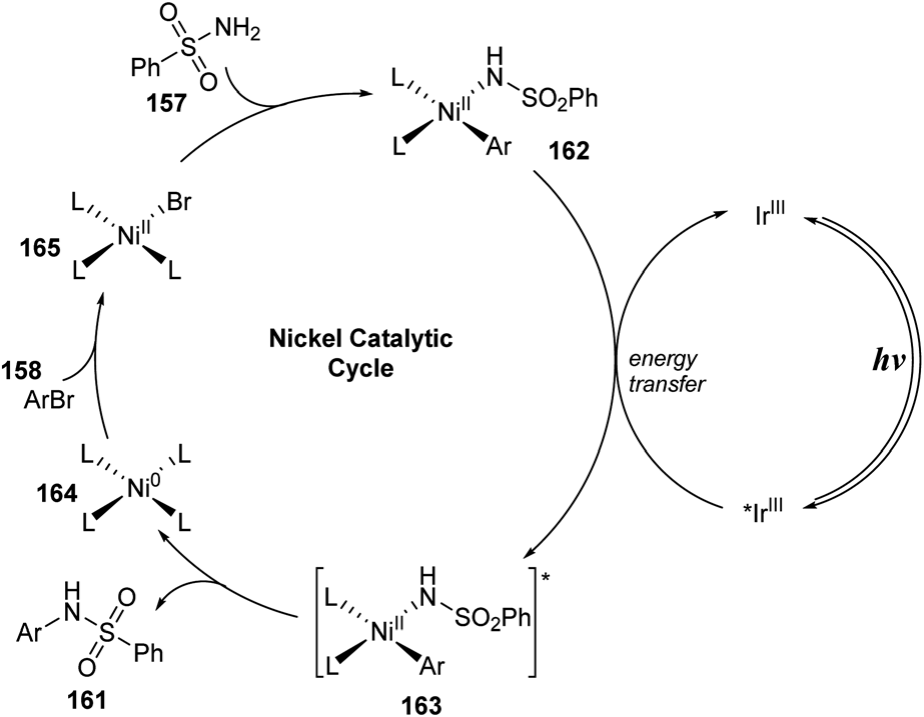
Proposed mechanism of sulfonamidation *via* photosensitized nickel-catalyzed cross-coupling.

A possible mechanism was proposed as shown in Scheme 43. The reaction between Ni^0^ complex 164 and aryl halide 158 formed Ni^II^-aryl complex 165, which directly interacted with sulfonyl amide 157 to give Ni^II^-aryl amido complex 162. At the same time, photocatalyst Ir^III^ in a singlet ground state was excited by visible light and was transferred to *Ir^III^ in a triplet excited state, which underwent an energy transfer process with complex 162 to yield intermediate 163 and ground state catalyst Ir^III^. Intermediate 163 rapidly decomposed to form *N*-aryl sulfonamide product 161 and return Ni^0^ complex 164.

**Scheme 44:**
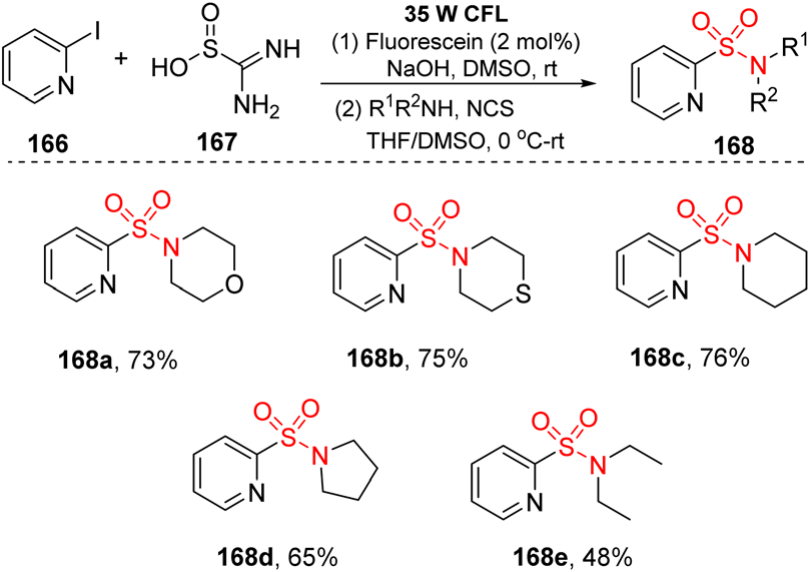
Sulfonamide synthesis from heteroaryl halides and thiourea dioxide.

Thiourea dioxide was employed as a SO_2_ surrogate instead of traditional sulfonyl sources such as DABSO, SO_2_ gas, Na_2_S_2_O_5_, or K_2_S_2_O_5_. In 2019, Li and co-workers reported novel visible-light-induced sulfonylation of heterocycles using thiourea dioxide.^95^ In this study, the reactions of (hetero)aryl halides and alkyl bromides to give (hetero)aryl sulfonyl alkyl compounds were conducted in the presence of fluorescein or Ir(ppy)_3_ as a photocatalyst and with NaOH in DMSO under irradiation of a compact fluorescent lamp (CFL) at room temperature (Scheme 44). Various heterocyclic sulfonamides were produced in good yields by this reaction system. Heterocyclic amines containing heteroatoms such as O and S were tolerated with this reaction, producing corresponding products (168a, 168b) with 73–75% yields. Additionally, cyclic amines with 5- or 6-member rings were transformed into the desired products (168c, 168d) in good yields. Straight-chain secondary amines as well as aniline derivatives were also compatible in this sulfonylation, even though the yields were not high.

**Scheme 45:**
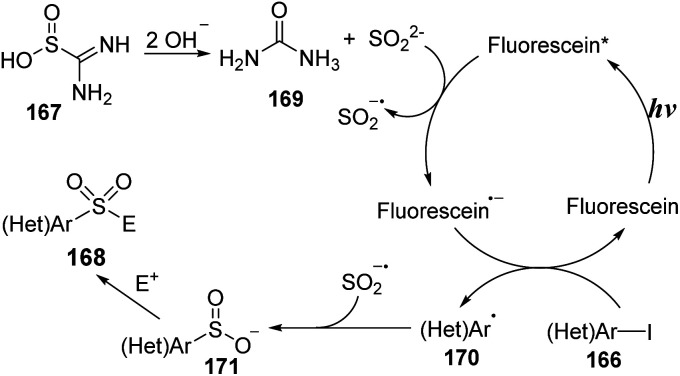
Plausible mechanism for sulfonamide synthesis from heteroaryl halides and thiourea dioxide.

A mechanism for the sulfonylation reaction was proposed as shown in Scheme 45. In the presence of base, thiourea dioxide 167 was decomposed to give sulfur dioxide anions and urea 169. Sulfur dioxide anions reacted with fluorescein*, which was excited from fluorescein by visible light, to form a sulfur dioxide radical anion and a fluorescein radical anion. Then, the fluorescein radical anions interacted with (hetero)aryl halide 166 to generate fluorescein ground state and heteroaryl radical 170, which combined with the sulfur dioxide radical anion to provide heteroaryl sulfinate intermediate 171. Finally, the heteroaryl sulfinate intermediate 171 reacted with electrophilic agents to generate the corresponding product 168.

**Scheme 46:**
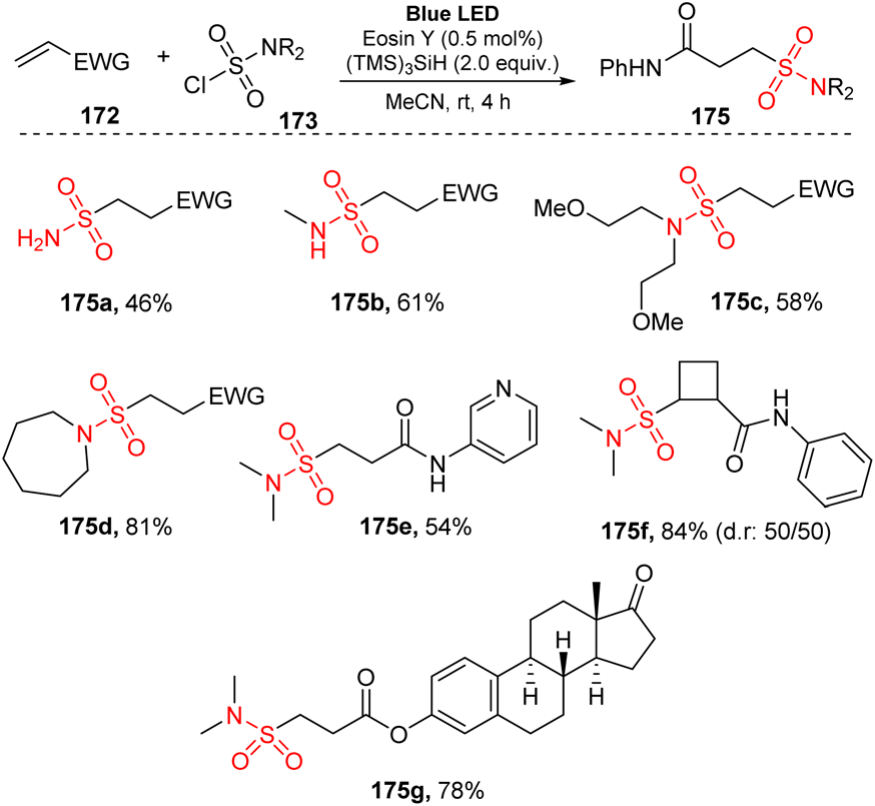
Hydrosulfamoylation of electron-deficient alkenes with sulfamoyl chlorides.

In 2020, Gouverneur used sulfamoyl chlorides for reaction with alkenes to synthesize alkyl sulfonamides.^96^ Reactions between alkenes and sulfamoyl chlorides were conducted in the presence of tris-(trimethylsilyl)silane ((TMS)_3_SiH) (as a silyl radical source and hydrogen atom donor) and eosin Y (as a photocatalyst) in MeCN under irradiation of a blue LED light at room temperature for 4 hours (Scheme 46). Various sulfamoyl chlorides including primary, secondary, and tertiary species were tolerated with this method to yield the corresponding alkyl sulfonamides (175a–175b) in 46–61% yields. Linear sulfonamides such as bis(2-methoxyethyl) sulfonylamide 175c were obtained with good yield under the standard reaction conditions. Cyclic sulfamoyl chloride was also compatible with this process, yielding the desired sulfonamide 175d in 81% yield. Additionally, diverse *N*-(hetero)arylacrylamides containing functional groups including electron-donating and electron-withdrawing groups on the (hetero)aryl ring were successfully employed in the reaction to provide the target product 175e in good yield. In this method, sulfamoyl chlorides bearing 1,2-disubstituted cyclobutene (175f), useful structures in medicinal chemistry, were smoothly prepared in good yields with a diastereomeric ratio of 50 : 50. In addition, an estrone-containing biologically active molecule underwent this reaction to yield the desired sulfonylamide (175g) in 78% yield.

**Scheme 47:**
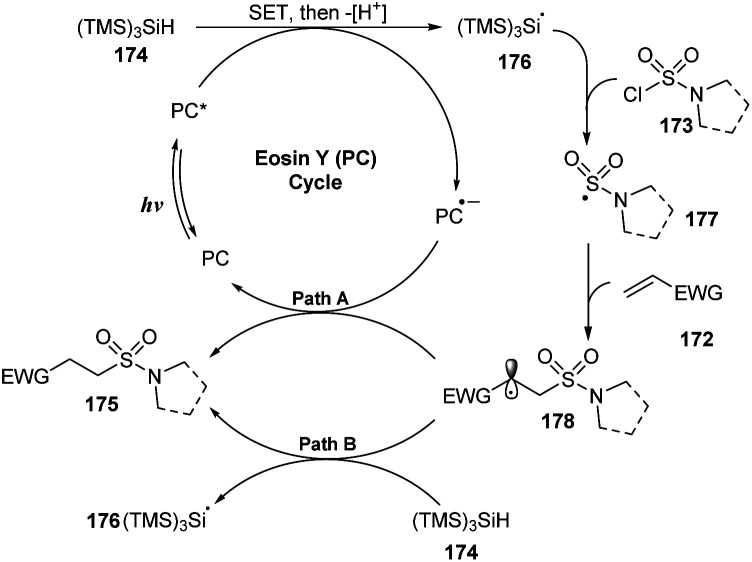
Photoredox-catalyzed hydrosulfamoylation of electron-deficient alkenes.

A proposed mechanism of this reaction is presented in Scheme 47. Visible light activated eosin Y (PC) to provide excited state PC*. A single-electron process was conducted between PC* and (TMS)_3_SiH 174 to give PC˙^−^ and (TMS)_3_SiH˙^+^, which then released one proton to form (TMS)_3_Si˙ radical 176. This radical captured one chloride atom of sulfamoyl chloride 173 to generate (TMS)_3_SiCl and sulfonyl amide radical 177. Radical 177 attacked the double bond of alkene 172 to provide alkyl radical 178. Another SET process between radical 178 and PC˙^−^ gave the hydrosulfamoylated product 175 and regenerated eosin Y. In a possible path, radical 178 also received one proton from (TMS)_3_SiH 174 to form the final product 175 and (TMS)3Si˙ radical 176.

**Scheme 48:**
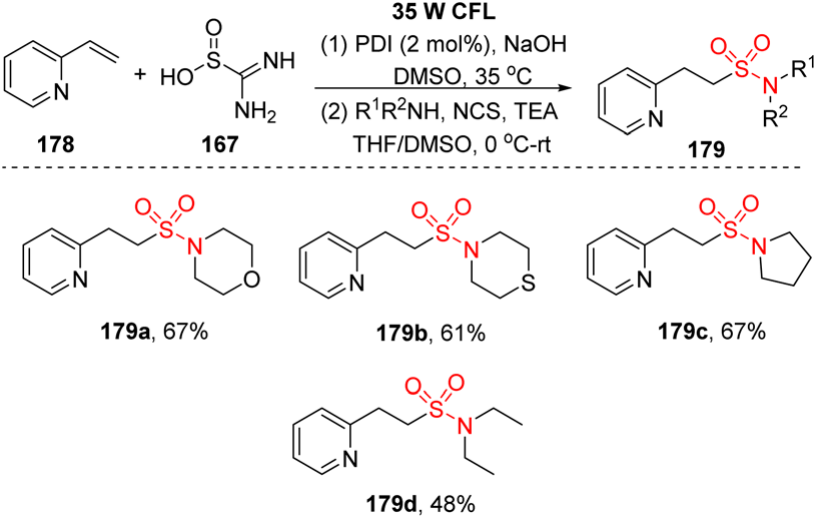
Synthesis of sulfonamides using alkenes, thiourea dioxide, and amines.

In a similar approach using thiourea dioxide as the sulfonyl source, Wu and co-workers performed sulfonylation of olefins to synthesize alkyl sulfonyl compounds including sulfones and sulfonamides.^97^ Heteroaryl olefins were reacted with thiourea dioxide and nucleophilic agents (bromide derivatives, amines) in the presence of perylene diimide (PDI) as a photocatalyst and NaOH in DMSO under radiation of a white compact fluorescent lamp (CFL) to form the corresponding products (Scheme 48). A wide range of alkyl sulfonamides was readily synthesized by this method using aliphatic amines as nucleophile agents and *N*-chlorosuccinimide instead of alkyl bromides. Most of the cyclic secondary amines were suitable for the reaction and afforded the corresponding products (179a–179c) with 61–67% yields. Open-chain amine provided a less effective outcome 179d (reaction yield of diethyl amine was 48%).

**Scheme 49:**
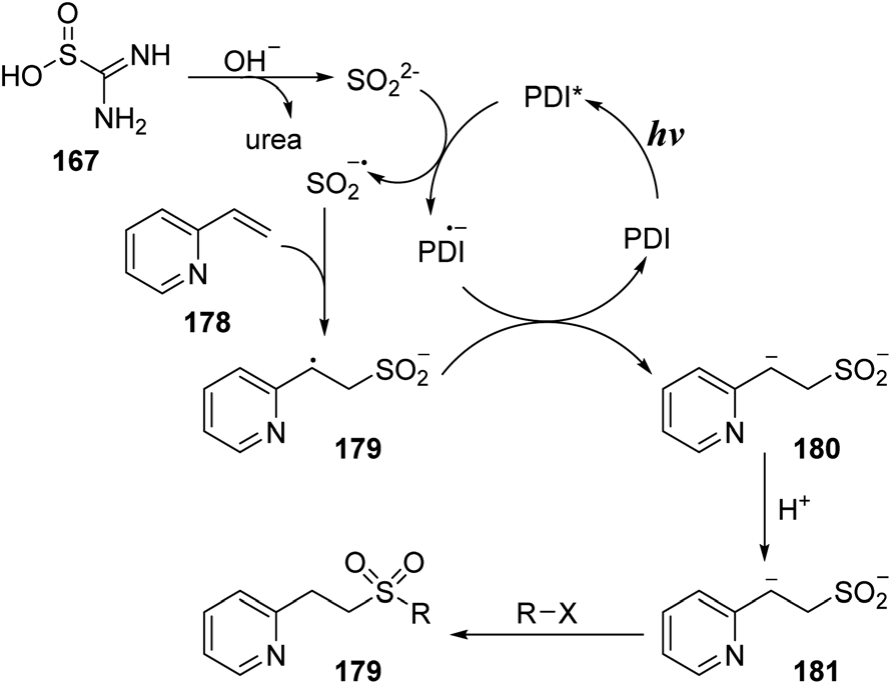
A plausible mechanism of sulfonylation using alkenes, thiourea dioxide and amines.

A reasonable mechanism has been proposed as shown in Scheme 49. Thiourea dioxide 167 reacted with base to yield SO_2_^2−^ anion and urea. Perylene diimide (PDI) photocatalyst in the ground state was excited by visible light to give PDI*, which would capture an electron from SO_2_^2−^ anion to provide SO_2_˙^−^ anion radical and PDI˙^−^ anion radical. The sulfonyl radical anion SO_2_˙^−^ underwent additional reaction with vinyl pyridine 178 to form intermediate 179, which received one electron from the PDI˙^−^ anion radical to generate intermediate 180 and returned PDI. At last, intermediate 180 was protonated to provide intermediate 181, which could react with many nucleophilic agents to provide the final product 179.

**Scheme 50:**
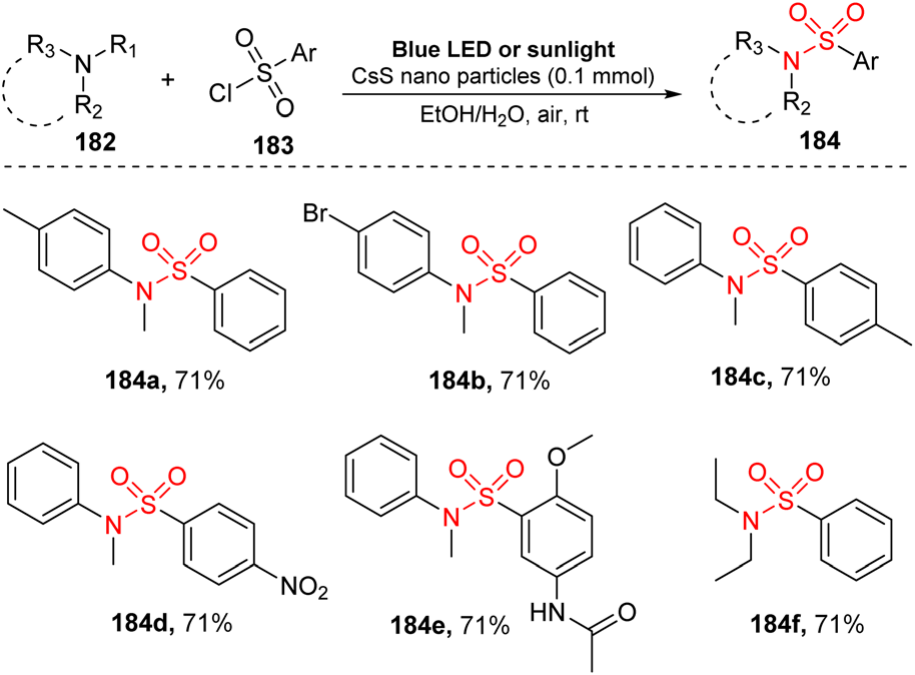
Sulfonylation reaction between tertiary amines and aryl sulfonyl chloride *via* an *N*-demethylation reaction using CdS NPs.

In 2021, Hosseini-Sarvari and co-workers used nanoparticles for visible-light-induced *N*-dealkylation of tertiary amines and aryl sulfonyl chloride (Scheme 50).^98^ Reactions were carried out in the presence of CdS nanoparticles as a photocatalyst in a mixture of EtOH and H_2_O under irradiation of blue LED (or sunlight) at room temperature under air. Various *N*,*N*-dialkylanilines bearing electron-withdrawing and electron-donating groups on the aromatic rings were well tolerated for this strategy to provide the desired sulfonamides (184a, 184b) in high yields. Similarly, diverse aryl sulfonyl chlorides with different functional groups were transformed smoothly into the corresponding products (184c–184e). Remarkably, several sulfonamides were prepared in a very short reaction time (10–15 minutes) using the CdS nanoparticle-catalyzed reaction.

**Scheme 51:**
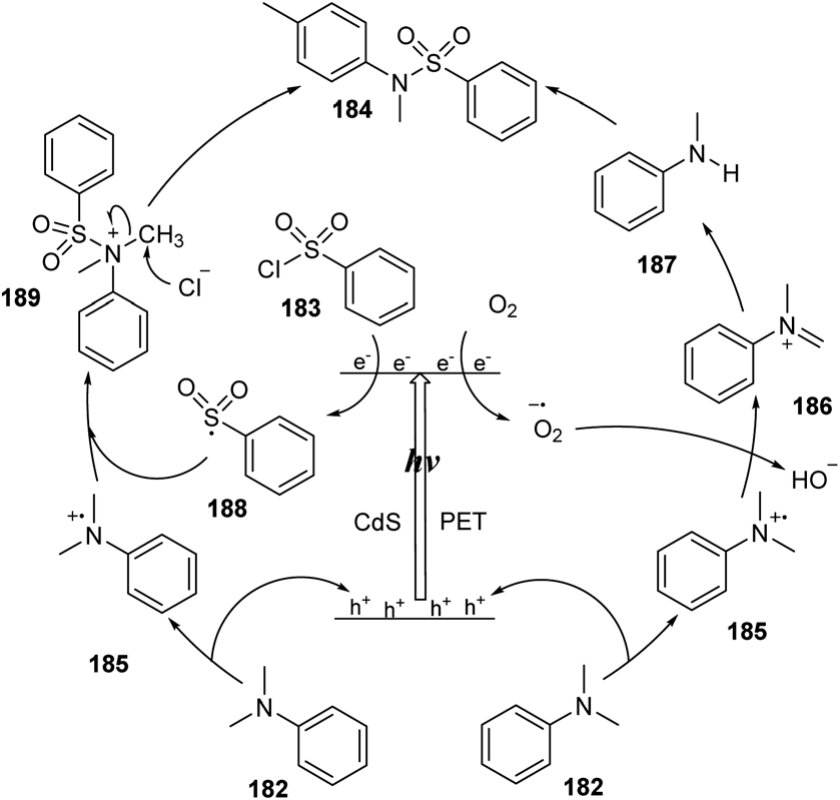
Proposed mechanism for sulfonylation between tertiary amines and aryl sulfonyl chloride *via N*-demethylation using CdS NPs.

A plausible mechanism is illustrated in Scheme 51. Under the visible light effect, electrons of CdS nanoparticles were excited and changed location from the valence band (VB) to the conduction band (CB), leading to the formation of holes (h^+^) and activated electrons (e^−^). Because of its lower oxidation potential, *N*,*N*-dimethylaniline 182 transferred electrons to holes (h^+^) of CdS nanoparticles to form radical cation 185. The activated electrons (e^−^) of CdS were trapped by oxygen O_2_ (or aryl sulfonyl chloride 183) to generate radical anion O_2_˙^−^ (or aryl sulfonyl radical 188 and chloride anion). Next, there were two pathways to produce sulfonamide products. In path I, radical cation 185 transferred one proton to radical anion O_2_˙^−^, producing anion HO_2_^−^ and immonium ion 186, which reacted with H_2_O to afford secondary amine 187. Reaction between 187 and sulfonyl chloride 185 generated the desired sulfonamide 184. In path II, radical cation 188 combined with aryl sulfonyl radical 185 to create cation 189 that reacted with chloride anion Cl^−^ to form the corresponding product 184 and CH_3_Cl.

**Scheme 52:**
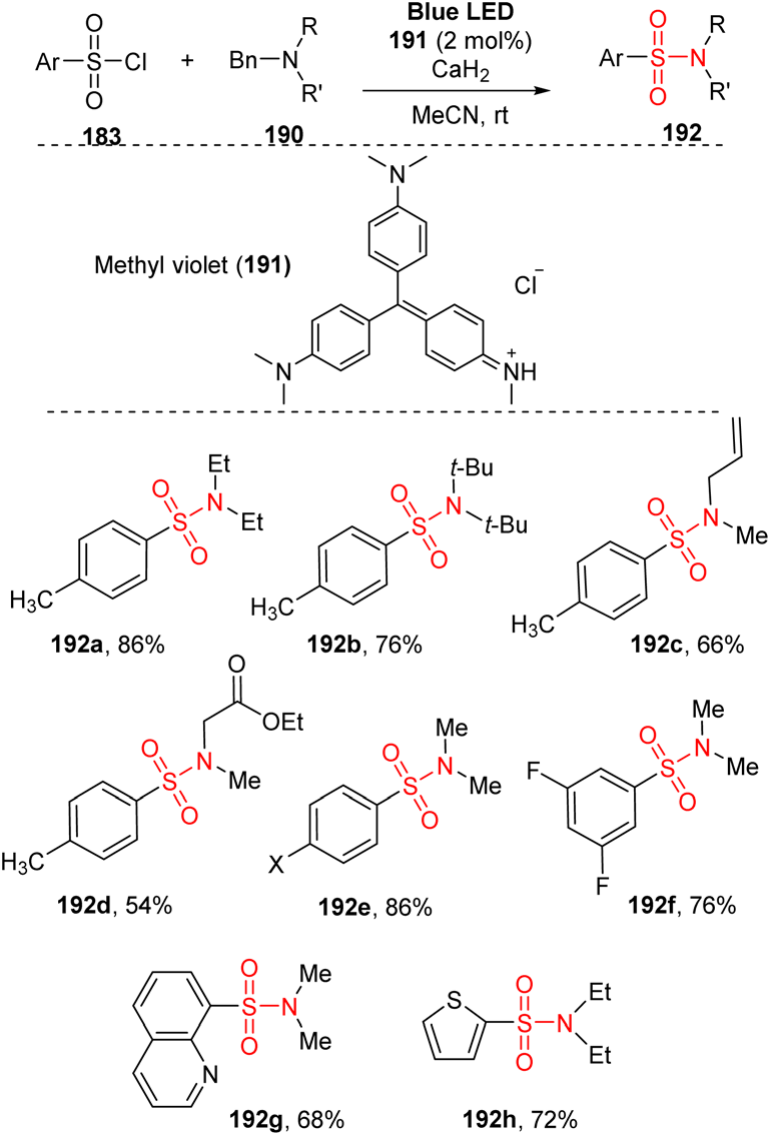
Photocatalytic debenzylative sulfonylation of tertiary benzylamines.

Tertiary alkylamines are rarely used as starting materials in chemical synthesis due to their stability and lack of the activity of N–H group. Tertiary alkylamines were employed to synthesize aryl sulfonyl amines by Fu and co-workers in 2021(Scheme 52).^99^ This method used aryl sulfonyl chloride derivatives and tertiary alkylamines as substrates, and the reactions were carried out in the presence of methyl violet as a photocatalyst and CaH_2_ as an additive in MeCN under irradiation of a blue LED. This method did not employ catalyst-coupling reactions using transition metals. Various tertiary benzylamines were tested for reactions of tosyl chloride to give the corresponding products (192a–192d) in good yields, even if they had sterically hindered substituents such as *N*,*N*-diisobutylbenzylamine or sensitive substituents such as allyl, ester, or ketone. Electron-poor and electron-rich aryl sulfonyl chlorides were readily converted to sulfonamide 192e in high yield. Additionally, a multi-substituent derivative was tolerated with this approach to give the desired product 192f with 76% yield. Heterocyclic sulfonyl chlorides were used in the standard reaction conditions, smoothly generating the target sulfonamide products (192g, 192h) in moderate yields.

**Scheme 53:**
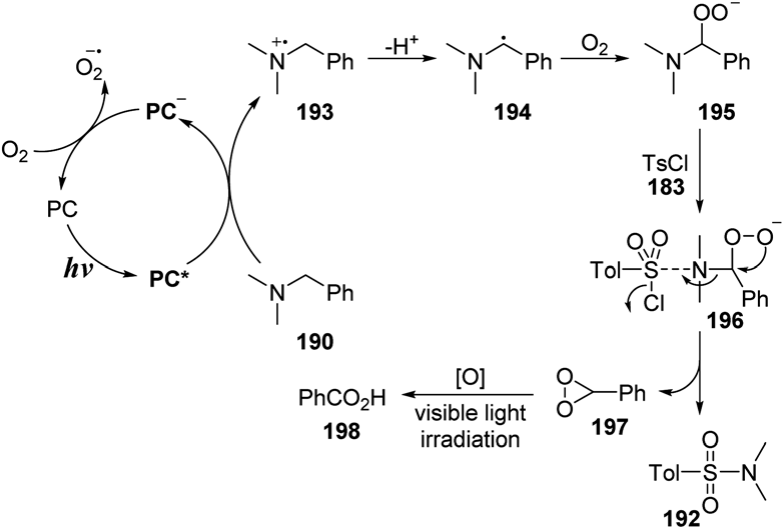
Proposed mechanism for photocatalytic debenzylative sulfonylation of tertiary benzylamines.

A proposed mechanism for this method is illustrated in Scheme 53. Under irradiation of a blue LED, methyl violet was transformed to excited state PC* and captured one electron from *N*,*N*-dimethylbenzylamine 190 to form PC˙^−^ anion radical and *N*-centered radical cation 193. After the hydro atom transfer (HAT) process and cross-coupled process between 193 and the perhydroxyl radical anion (O_2_˙^−^), the intermediate 193 was converted to peroxide 195. Then, peroxide 195 was reacted with aryl sulfonyl chloride 183 to form complex 196. This complex 196 underwent the electron transfer process and was decomposed to the sulfonamide product 192, as well as an unstable 3-phenyldioxirane intermediate 197, which was oxidized to benzoic acid under irradiation of visible light. Separately, the PC˙^−^anion radical reacted with O_2_ to return ground state PC to provide a reactive perhydroxyl radical anion (O_2_˙^−^).

**Scheme 54:**
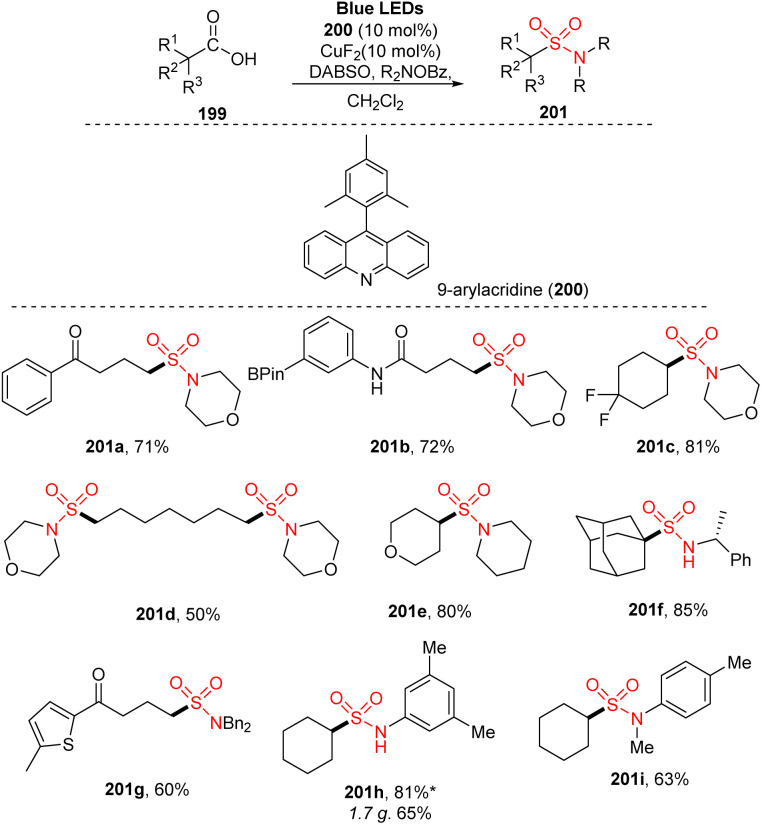
Direct decarboxylative amidosulfonation with *O*-benzylhydoxylamines and aniline.

In 2021, Larionov and co-workers performed direct functionalization of a carboxylic acid to afford sulfonamides and sulfonyl azides *via* a multicomponent reaction (Scheme 54).^100^ A dual catalytic system of copper salts and 9-aryl acridine derivative was employed for reactions of carbocyclic acid in the presence of copper salts and 9-aryl acridine as catalysts, DABSO as a SO_2_ supplier, and *O*-benzoylhydroxylamine or amine in CH_2_Cl_2_ under irradiation of a blue LED under mild conditions. Various straight-chain aliphatic carboxylic acids with different substituent groups (including electron-rich aryl and boryl groups) were well tolerated in the reaction with *O*-benzoyl hydroxyl morpholine to produce sulfonamides (201a, 201b). A wide range of cyclic acids was successfully converted to the corresponding products 201c. Target product bearing two sulfonamide groups were also smoothly prepared (201d, 50%). In addition, when amines other than morpholine were used, reactions were smoothly carried out to give the desired products (201e–201g) with good yields (60–85%). Reactions of cyclohexane carboxylic acid with aniline derivatives such as *p*-, *m*-, and *o*-substituted anilines (201h, 201i) were successfully carried out. Several reactions using combinations of acids and anilines were tested, and the target products were readily obtained in good yields.

**Scheme 55:**
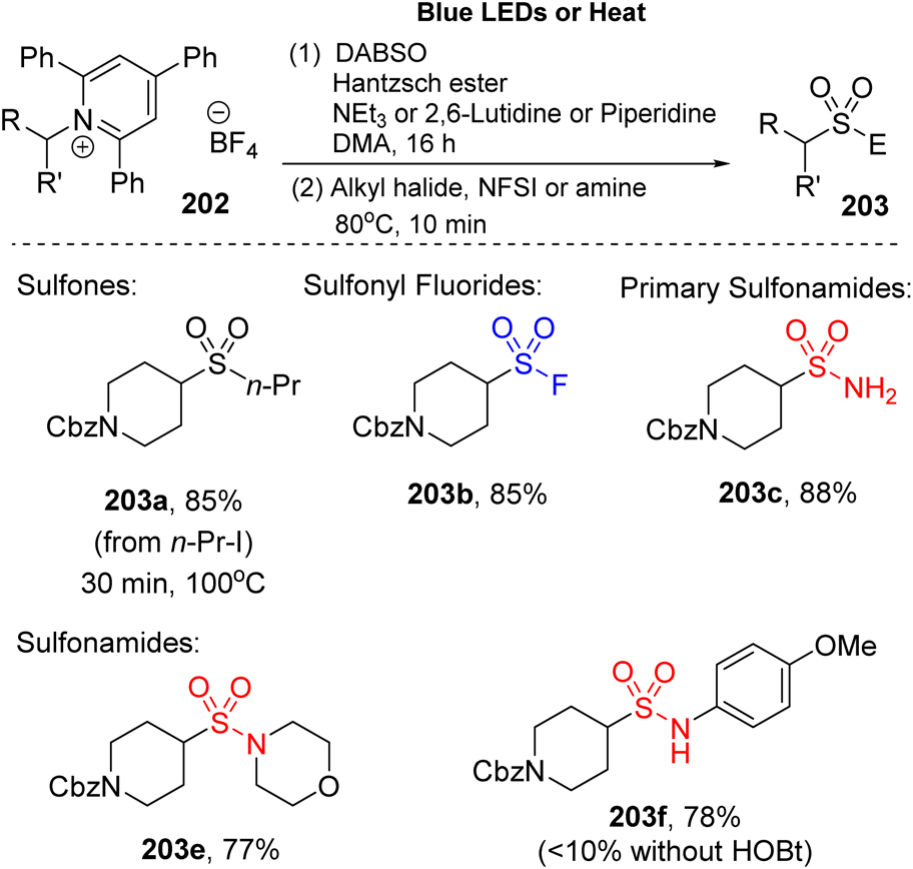
Synthesis of sulfonyl compounds using Katritzky salt.

2,4,6-Trisubstituted pyridinium salt (Katritzky salt) were employed to synthesize alkyl sulfonyl derivatives by Willis and co-workers because Katritzky salt generates alkyl radicals under mild conditions (Scheme 55).^101^ The reaction consisted of two steps. Katritzky salt was reacted with DABSO and Hantzsch ester in the presence of a base (such as triethylamine, 2,6-lutidine, or piperidine) in DMA under irradiation of a blue LED, followed by alkylation with various electrophile agents to afford the products. A wide range of carbon-electrophiles was utilized in this method to give the target products. When an alkyl halide or NFSI rather than *t*-butylbromoacetate was used, nonsymmetric sulfones (203a) and sulfonyl fluoride (203b) were easily obtained with high yields. In addition, primary and secondary amines were rapidly converted to the corresponding products (203c, 203e) (88–77%). The aniline derivatives are also suitable for this reaction and generated the sulfonamides (203f) with good yield (78%).

**Scheme 56:**
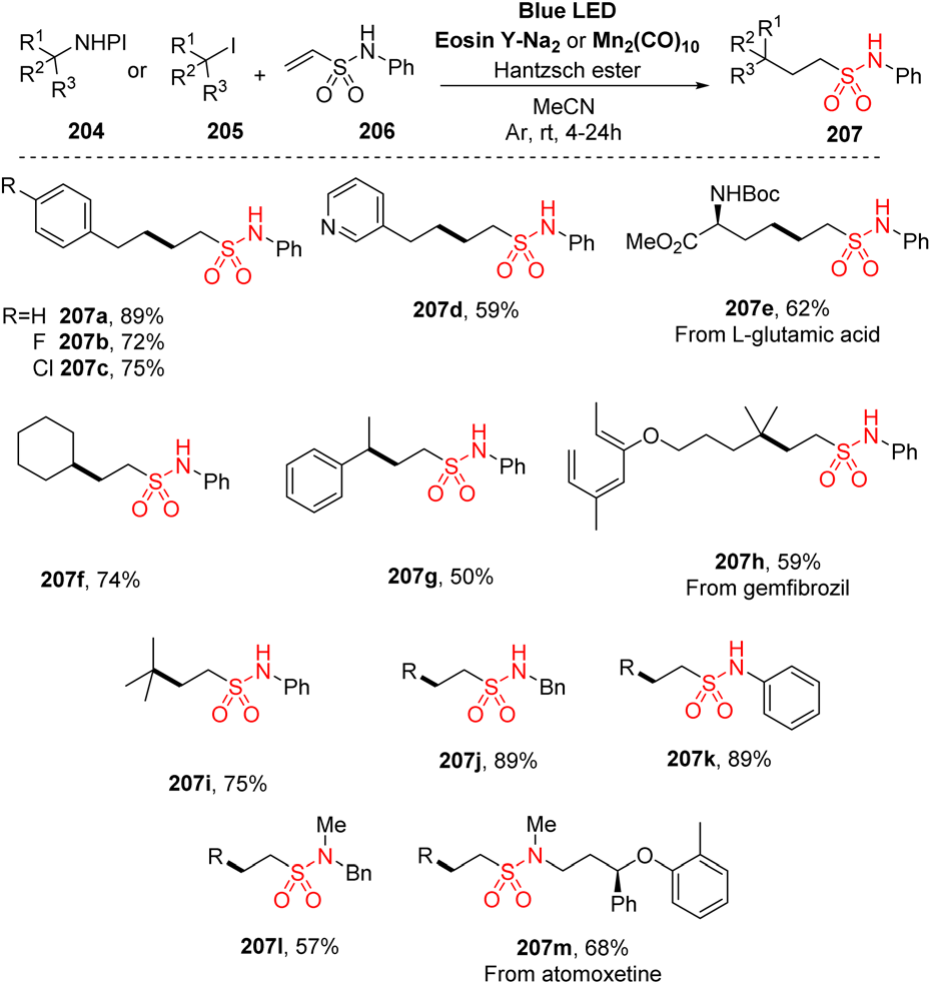
Synthesis of sulfonamides using alkyl carboxylic acids, alkyl iodides, and vinyl sulfonamide.

Recently, Wang and co-workers reported a novel efficient approach to synthesize aliphatic sulfonamides by employing vinyl sulfonamides as radical acceptors (Scheme 56).^102^*N*-Hydroxyphthalimide esters or alkyl iodides were used as alkyl radical sources in a reaction with *N*-phenylethenesulfonamides. The reactions were conducted in the presence of eosin Y salt as a photocatalyst and Hantzsch ester as an additive in MeCN under irradiation of blue LED light. Various alkyl radicals from *N*-hydroxyphthalimide esters or alkyl iodides were investigated. 3-Phenylpropanoic acid and its derivatives including electron-withdrawing or electron-donating groups were smoothly reacted with *N*-phenylethenesulfonamide to yield the corresponding products (207a–207c) in 56–86% yields. Reaction of primary alkyl substrates with functional groups such as *N*-heterocycles at the end chain or with Boc-protected amine generated the desired sulfonamides (207d and 207e) in acceptable yield. Similarly, secondary (207f, 207g) and tertiary (207h, 207i) alkyls were successfully used in the operations, yielding the target products. A wide range of amines was reacted with NHPI ester to prepare vinyl sulfonamides. Reactions of primary amines such as benzyl amine or aniline gave the corresponding products (207j and 207k) with high yields, while secondary amines delivered product with lower yield (207l). This protocol was also smoothly applied to late-stage functionalization to synthesize medicines and natural products (207m).

**Scheme 57:**
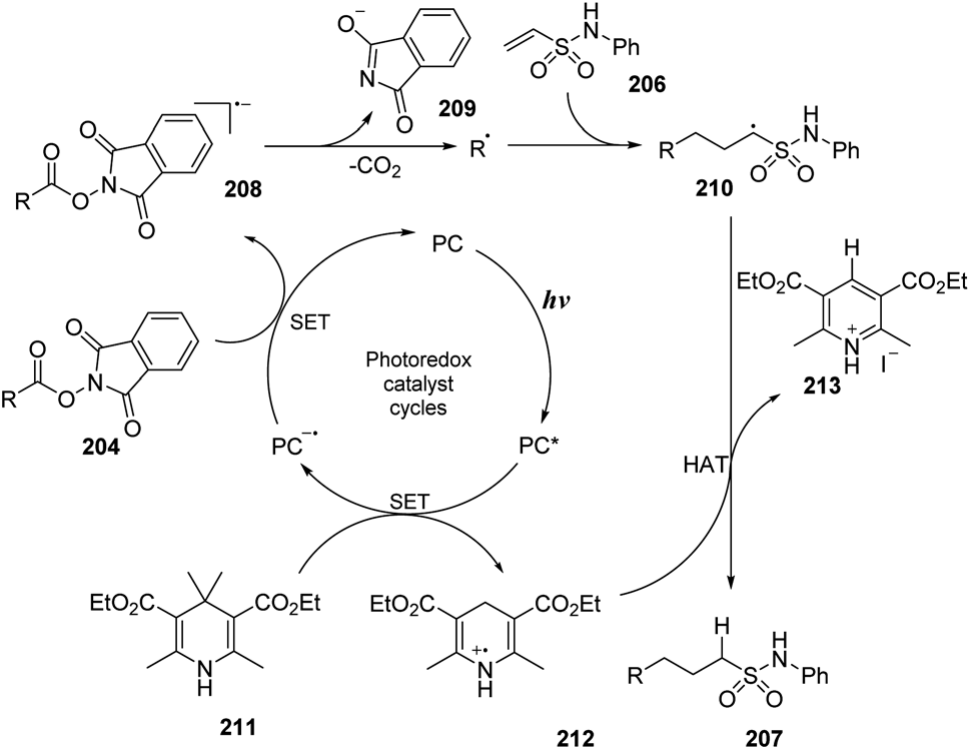
Proposed mechanism for synthesis of sulfonamides using *N*-hydroxyphthalimide esters or alkyl iodides.

A proposed mechanism of the reaction is shown in Scheme 57. Eosin Y (PC) was transformed into the excited state under irradiation of light. Then, it underwent the SET process with Hantzsch ester 211 to generate radical cation 212 and radical anion PC˙^−^. The PC˙^−^ radical anion underwent another SET process with ester substrate 204 to form radical anion 208 and returned a ground state photocatalyst. The intermediate 208 was quickly decomposed by cleavage of the N–O bond to afford alkyl radical, CO_2_, and anion 209. In the last step, the alkyl radical attacked the double bond of vinyl sulfonamide 206 to generate intermediate radical 210, which captured a proton from radical cation 212 to form the desired product 207.

## 3. Conclusions

Sulfonylation is important because sulfur dioxide-containing compounds have been widely employed in chemistry, pharmaceuticals, and biological processes. In this review, we summarized recent developments in photochemical reactions to synthesize sulfonyl fluorides, sulfonic esters, and sulfonyl amides.

The reported sulfonylation strategies described discoveries of novel substrates including sources of sulfonyl group and acceptor compounds to generate valuable compounds. Various studies for development of novel synthetic methods with reducing additives or reducing demanding requirements for the reactions were also carried out. In the studies presented, most of the sulfonylation processes were carried out under visible or UV light, with the support of different photocatalysts, depending on the reaction. According to the proposed reaction mechanisms, SET in photochemical reactions takes place throughout the transformation either in an oxidative or reductive fashion. Unfortunately, it is still necessary to use catalysts that are transition metals, or expensive photocatalysts, as well as special additives to perform SET or oxidation processes for synthesis. These requirements have reduced the greenness and increased the cost of the synthesis processes and limited the applicability of the method.

In recent research, scientists reported direct synthesis processes that did not use metal catalysts or that used photocatalysts that have overcome the above disadvantages, but there are few of these studies.^80,89,93,101^ Therefore, there are many opportunities to develop new synthetic methods using visible light to synthesize sulfur dioxide-containing compounds. Discovery of sulfonylation reactions conducted under light without transition metal or photocatalysts as well as identification or creation of new sulfonyl sources and precursors will provide more effective synthesis methods for high-value structures in the future.

## Author contributions

Conceptualization, H.-K. K.; writing—original draft preparation, T. G. L. and H.- K. K.; writing—review and editing, T. G. L. and H.-K. K.; funding acquisition, H.-K. K. All authors have read and agreed to the published version of the manuscript.

## Conflicts of interest

There are no conflicts to declare.

## Supplementary Material
